# Functional illiteracy burden in soil-transmitted helminth (STH) endemic regions of the Philippines: An ecological study and geographical prediction for 2017

**DOI:** 10.1371/journal.pntd.0007494

**Published:** 2019-06-21

**Authors:** Kei Owada, Mark Nielsen, Colleen L. Lau, Laith Yakob, Archie C. A. Clements, Lydia Leonardo, Ricardo J. Soares Magalhães

**Affiliations:** 1 School of Medicine, The University of Queensland, South Brisbane, Queensland, Australia; 2 Children’s Health and Environment Program, Child Health Research Centre, The University of Queensland, South Brisbane, Queensland, Australia; 3 Spatial Epidemiology Laboratory, School of Veterinary Science, The University of Queensland, Gatton, Queensland, Australia; 4 School of Psychology, The University of Queensland, St Lucia, Queensland, Australia; 5 Faculty of Humanities, University of Johannesburg, Auckland Park, South Africa; 6 Research School of Population Health, Australian National University, Canberra, Australian Capital Territory, Australia; 7 Department of Disease Control, London School of Hygiene and Tropical Medicine, London, United Kingdom; 8 Department of Parasitology, College of Public Health, University of the Philippines Manila, Manila, Philippines; McGill University, CANADA

## Abstract

**Background:**

Soil-transmitted helminth (STH) infections remain highly endemic across the Philippines, and are believed to be important contributors to delayed cognitive development of school-aged children. Identification of communities where children are at risk of functional illiteracy is important for the attainment of Sustainable Development Goals target for literacy. We aimed to quantify the associations between the spatial variation of STH infections and functional literacy indicators adjusting for other important contributors, and identify priority areas in the Philippines in need of interventions.

**Methodology/Principal findings:**

We used data from 11,313 school-aged children on functional literacy indicators collected in 2008. Nested fixed-effects multinomial regression models were built to determine associations between STH endemicity and geographical distribution of functional literacy, adjusting for demographics, household level variables, and the prevalence of malaria. Bayesian multinomial geostatistical models were built to geographically predict the prevalence of each level of functional literacy. The number of school-aged children belonging to each of the functional literacy indicator classes was forecast for 2017.

We estimated 4.20% of functional illiteracy burden among school-aged children in Mindanao might be averted by preventing *T*. *trichiura* infections. Areas predicted with the highest prevalence of functional illiteracy were observed in localised areas of the eastern region of the Visayas, and the south-eastern portion of Mindanao.

**Conclusions/Significance:**

The study demonstrates significant geographical variation in burden of functional illiteracy in school-aged children associated with STH infections suggesting that targeted helminth control could potentially promote the development of cognitive function of school-aged children in the Philippines. The benefits of a spatially targeted strategy should be tested by future studies.

## Author summary

While previous studies in the Philippines indicated an association between STH infection and cognitive development measured by memory and school performance, the contribution of STH infections on the overall functional illiteracy burden in the Philippines is unknown. This study presents the first use of geographical risk models of functional literacy adjusted for a wide array of probable confounders to uncover the associations with STH infections. This study also explores how the application of spatial epidemiology in mapping functional illiteracy provides an evaluation-planning tool for the design and implementation of STH-associated morbidity control intervention strategies, estimating the number of school-aged children with functional illiteracy associated with STH infections, and the number of interventions needed in the Philippines.

## Introduction

Functional literacy is one of the targets of the Sustainable Development Goals (SDG) of the United Nations, launched in September 2015 [1]. Functional literacy is a key indicator of cognitive function, especially information processing and comprehension, and it has been used to measure cognitive function in school-aged children [2]. According to the latest United Nations Educational Scientific and Cultural Organization report on literacy, there are globally 114 million illiterate adolescents and youths [3]. Despite widespread acknowledgement of this problem, between 2000 and 2015, global literacy rates were estimated to have improved by just 4%. Of particular relevance to the current work, progress in addressing national literacy rates in the Philippines has been slow [4].

Debate has recently intensified regarding the role of soil-transmitted helminths (STH), *Ascaris lumbricoides*, *Trichuris trichiura*, and hookworms (*Ancylostoma duodenale*, *A*. *ceylanicum* and *Necator americanus*) in childhood cognitive function, and by extension functional literacy [5]. To date, relatively few studies have investigated this link and evidence remains inconclusive, partly because differences in study designs and the use of cognitive development measurement tools makes it difficult to compare results of studies [6]. STH infections are among the most common infections in school-aged populations, and are particularly common in impoverished communities where the provision of water, sanitation, and hygiene education is limited [7]. STH infections are estimated to incur 4.98 million years lived with disability, related to anaemia, chronic nutritional imbalances, stunting, and cognitive and motor developmental delay [8].

Possible mechanisms for the effect of STH on cognitive function in children may include the interaction between the host immune system and the species of STH, the direct effect of metabolites excreted during infection, anaemia, or inflammation, and through secondary effects such as malnutrition and micronutrient deficiencies [9]. A recent experimental study demonstrated that *T*. *trichiura* infection may influence cognitive function of animals, although a definitive prospective human study has not been performed [10].

STH infections remain highly endemic across the Philippines among both primary and secondary school children [11, 12]. While previous studies in the Philippines indicated an effect of STH infection on children’s cognitive development, the contribution of STH infections on the overall functional illiteracy burden in the Philippines is unknown [13–15].

Processes that result in reduced functional literacy are complex and multifactorial. Compromised nutritional and family contextual factors such as poverty, unemployment, low maternal education, and household education stimuli have been linked consistently with cognitive function and educational performance of school-aged children [16]. In developing countries such as the Philippines, cognitive dysfunction could be caused by malnutrition in complex combinations with other factors including deprivation of environmental and emotional stimulation, biological factors, and infections such as pneumonia, meningitis, STH, and malaria [17].

Predictive risk maps, together with geospatial modelling, are emerging as important decision tools for public health policy makers [18]. However, to date there have been no studies looking at geographical variation in functional literacy and the associations with its key determinants.

In this study, we aimed to quantify the associations between the spatial variation of STH infections and functional literacy in school-aged children in the Philippines, adjusting for probable confounders. In doing so, we also aimed to develop the first prediction maps of each functional literacy indicator in order to quantify the number of school-aged children at risk of reduced functional literacy in the Philippines.

## Materials and methods

### Ethics statement

Ethical clearance for this analytical study was provided by the University of Queensland School of Medicine Low Risk Ethical Review Committee (clearance number 2014-SOMILRE-0100).

### Study design and data

We obtained population level data on functional literacy indicators of 11,313 school-aged children (aged 10–19 years) collected in 2008 during the nationwide Functional Literacy, Education and Mass Media Survey (FLEMMS). In brief, a total of 1,506 barangays (the smallest administrative unit in the Philippines: average diameter of 11 km) were included, 851 located in Luzon, 254 in the Visayas, and 401 in Mindanao (A map of 2008 FLEMMS survey locations is shown in S1 Fig [19]). All data analysed were anonymised.

Functional literacy levels were stratified into four classifications – 1) those who cannot read, write, compute and comprehend were classified as functional illiterate; 2) those who can only read and write (with understanding a simple message) were considered as low functional literate; 3) those who can read, write and compute were considered as moderate functional literate; and 4) those who can read, write, compute and comprehend (with a significantly higher level of literacy that includes not only reading and writing skills but also numerical and comprehension skills) were considered as functional literate. Further detailed information on the FLEMMS can be found in S1 Text.

A wide array of plausible contributing factors were obtained from the FLEMMS individual questionnaires and FLEMMS household questionnaires, including individual level sociodemographic indicators (e.g. age, sex, education attainment level, marital status, and employment status), and household level factors such as socioeconomic status (SES), access to water, sanitation, and hygiene (WASH), and household education stimuli. Information regarding head of households such as adult functional literacy was measured for a total of 10,339 heads of households. We obtained data of spatial predicted values of *Plasmodium* infections from the Malaria Atlas Project (S2 Fig) [20]. We used predictive maps of STH infection prevalence generated from spatial analysis of the data collected during the National Schistosomiasis Survey in the Philippines in 2005 to 2007 (S3 and S4 Figs) [12, 21]. Further information on each indicator is detailed in S2 Text.

### Analysis

#### Geo-referencing of data

The unit of analysis was the barangay. Functional literacy survey data were linked to barangay centroids (longitude and latitude; geographical unit of analysis), which were estimated using the geographical information system (GIS) software Quantum GIS version 1.7.3 [22] based on a shapefile of the barangays of the Philippines, obtained from the geographic data warehouse DIVA GIS (http://www.diva-gis.org/Data) and PhilGIS (https://www.philgis.org). We extracted predicted values of STH infection profiles and malaria endemicity for each FLEMM survey location in ArcGIS version 10.4.0.5524 [23].

#### Multinomial logistic regression models

The prevalence of functional literacy levels was modelled by transforming the original 2008 FLEMMS functional literacy levels into an ordinal variable. Functional literacy was used as a reference group in all models. Five nested frequentist fixed-effects multinomial regression models (Tables 1–3) were developed for Luzon, the Visayas and Mindanao separately. We used Akaike’s Information Criterion (AIC) to choose the best-fitted model. For Luzon and the Visayas, Model 3 fits the data best (lowest AIC), whereas for Mindanao, Model 5 provided the best fit to the data. In all models, we report ratio of relative risks (RRR) for each variable in the multinomial logistic regression, adjusted for the range of candidate predictors. Additionally, we developed Model 6 to also include *T*. *trichiura* infection intensity classes for Mindanao (Table 4). All analyses were conducted in STATA version 13.1 [24]. Further information on our multinomial logistic regression models, variable selection, collinearity, and interaction assessment are detailed in S3 Text.

**Table 1:** List of covariates included in the models, Luzon.

Models	Sociodemographic: age, sex, education, adult literacy rate	Socioeconomic status (SES)	Water, sanitation and hygiene (WASH)	Household education stimuli	Prevalence of *Pf*PR_2-10_	Prevalence of *Pv*PR_2-10_	Prevalence of *A*. *lumbricoides*	Prevalence of *T*. *trichiura*	Prevalence of Hookworm	Prevalence of mono- and coinfection of *A*. *lumbricoides* and *T*. *trichiura*	AIC
**No infection**	✓	✓	✓	✓	✗	✗	✗	✗	✗	✗	10,437.38
**Model 1**	✓	✓	✓	✓	✓	✓	✗	✗	✗	✗	3,468.11
**Model 2**	✓	✓	✓	✓	✓	✓	✓	✗	✗	✗	3,467.63
**Model 3**	✓	✓	✓	✓	✓	✓	✓	✓	✗	✗	3,460.24
**Model 4**	✓	✓	✓	✓	✓	✓	✓	✓	✓	✗	3,464.34
**Model 5**	✓	✓	✓	✓	✓	✓	✗	✗	✗	✓	3,463.09

**Table 2:** List of covariates included in the models, the Visayas.

Models	Sociodemographic: age, sex, education, adult literacy rate	Socioeconomic status (SES)	Water, sanitation and hygiene (WASH)	Household education stimuli	Prevalence of *Pf*PR_2-10_	Prevalence of *Pv*PR_2-10_	Prevalence of *A*. *lumbricoides*	Prevalence of *T*. *trichiura*	Prevalence of Hookworm	Prevalence of mono- and coinfection of *A*. *lumbricoides* and *T*. *trichiura*	AIC
**No infection**	✓	✓	✓	✓	✗	✗	✗	✗	✗	✗	3,517.26
**Model 1**	✓	✓	✓	✓	N/A	N/A	✗	✗	✗	✗	3,517.26
**Model 2**	✓	✓	✓	✓	N/A	N/A	✓	✗	✗	✗	3,515.42
**Model 3**	✓	✓	✓	✓	N/A	N/A	✓	✓	✗	✗	3,473.16
**Model 4**	✓	✓	✓	✓	N/A	N/A	✓	✓	✓	✗	3,511.25
**Model 5**	✓	✓	✓	✓	N/A	N/A	✗	✗	✗	✓	3,486.21

Note: N/A = Not available.

**Table 3:** List of covariates included in the models, Mindanao.

Models	Sociodemographic: age, sex, education, adult literacy rate	Socioeconomic status (SES)	Water, sanitation and hygiene (WASH)	Household education stimuli	Prevalence of *Pf*PR_2-10_	Prevalence of *Pv*PR_2-10_	Prevalence of *A*. *lumbricoides*	Prevalence of *T*. *trichiura*	Prevalence of Hookworm	Prevalence of mono- and coinfection of *A*. *lumbricoides* and *T*. *trichiura*	AIC
**No infection**	✓	✓	✓	✓	✗	✗	✗	✗	✗	✗	5,077.03
**Model 1**	✓	✓	✓	✓	✓	✓	✗	✗	✗	✗	2,724.77
**Model 2**	✓	✓	✓	✓	✓	✓	✓	✗	✗	✗	2,703.08
**Model 3**	✓	✓	✓	✓	✓	✓	✓	✓	✗	✗	2,708.38
**Model 4**	✓	✓	✓	✓	✓	✓	✓	✓	✓	✗	2,701.12
**Model 5**	✓	✓	✓	✓	✓	✓	✗	✗	✗	✓	2,700.54

**Table 4:** List of covariates included in the models, Mindanao (infection intensity classes).

Models	Sociodemographic: age, sex, education, adult literacy rate	Socioeconomic status (SES)	Water, sanitation and hygiene (WASH)	Household education stimuli	Prevalence of *Pf*PR_2-10_	Prevalence of *Pv*PR_2-10_	Prevalence of light infection intensity class	Prevalence of moderate/high infection intensity class	AIC
**No infection**	✓	✓	✓	✓	✗	✗	✗	✗	5,077.03
**Model 6**	✓	✓	✓	✓	✓	✓	✓	✓	2,688.53

#### Estimation of the population attributable fraction

We calculated the population attributable fraction (PAF) using the standard formula to quantify the contribution of risk factors to functional illiteracy. Further information on the PAF estimation are detailed in S4 Text.

#### Analysis of spatial dependence

Residuals for final non-spatial models were extracted and examined for spatial autocorrelation by generating semivariograms using the geoR package in the R software version 3.1.1 (http://www.R-project.org). A semivariogram is a graphical representation of the spatial variation in a dataset. In the case of residual semivariograms, these represent the spatial variation left unexplained by the covariates included in a model.

#### Spatial risk prediction

Multinomial spatial models, including an intercept, a geostatistical random effect, and the infection variables selected based on the best-fitted model in our non-spatial models were built for each region of the Philippines using OpenBUGS version 3.2.3 [25]. Model predictions were used to generate representative risk maps of the prevalence of each class of functional literacy across the Philippines using ArcGIS version 10.4.0.5524. Further information regarding model specification and prediction maps is provided in S5 and S6 Text, respectively.

#### Model validation

The area under the curve (AUC) of the receiver operating characteristic was used to quantify the discriminatory performance of the model relative to observed mean prevalence threshold at validation locations. In the case of moderate functional literacy, we used a threshold of 20% for all regions; in the case of low functional literacy 5% for Luzon and Mindanao, and 7% for the Visayas; and in the case of functional illiteracy 3% for Luzon, 7% for the Visayas, and 6% for Mindanao. An AUC of $0.50-0.6\bar{9}$ was taken to indicate poor discriminative ability, $0.70-0.8\bar{9}$ reasonable discriminative ability, and ≥0.90 good discriminatory ability [26].

#### Estimation of the number of school-aged children in each functional literacy class in 2017

We multiplied each of the predictive raster maps of functional literacy indicators by a raster map of the estimated total number of school-aged children (people per square kilometre) in 2017. The forecast for 2017 is based on a constant trend in the prevalence of selected functional literacy profiles. Further information regarding estimation of the number of school-aged children belonging to each of the functional literacy indicators is provided in S7 Text.

## Results

A total of 11,313 school-aged children and 10,339 heads of household with complete information were included in the analysis. The highest prevalence of functional illiteracy in school-aged children was observed in the Visayas, followed by Mindanao and Luzon (7.5%, 6.9%, and 3%, respectively; Table 5). The observed prevalence of functional illiteracy of heads of households was also higher in the Visayas and Mindanao compared to Luzon (15%, 15%, and 6%, respectively; S2 Table). Full descriptive results of the dataset are presented in S3 and S4 Tables, and S5 Fig [19].

**Table 5:** Demographic characteristics of children, stratified by regions of the Philippines.

	Variables	Regions		
		Luzon (n = 6,616)	The Visayas (n = 1,814)	Mindanao (n = 2,883)
**Age**	Mean age (Standard deviation)	13.5 (2.37)	13.6 (2.46)	13.8 (2.50)
**Sex**	Male	3,427 (51.8)	924 (50.9)	1,485 (51.5)
	Female	3,189 (48.2)	890 (49.1)	1,398 (48.5)
**Functional literacy**	Functional literate	4,544 (68.7)	1,001 (55.2)	1,584 (54.9)
	Moderate functional literate	1,506 (22.8)	532 (29.3)	941 (32.6)
	Low functional literate	366 (5.5)	145(8.0)	159 (5.5)
	Functional illiterate	200 (3.0)	136 (7.5)	199 (6.9)
**Highest education attainment**	No grade completed	33 (0.5)	24 (1.3)	58 (2.0)
	Elementary school level	3,765 (56.9)	1,079 (59.5)	1,730 (60.0)
	Junior high school level	2,818 (42.6)	711 (39.2)	1,095 (38)
**Employment status**	Yes	689 (10.0)	314 (17.3)	472 (16.4)
	No	5,927 (90.0)	1,500 (82.7)	2,411 (83.6)

Note: Unless otherwise indicated, values represent the absolute number, followed by the percentage within parentheses.

While the relative importance of determinants of functional literacy varied between regions, some findings were consistent across all regions (Table 6). We found that highest education attainment, low socioeconomic status (SES) and adult functional illiteracy rates are major contributors to functional illiteracy across Luzon, the Visayas and Mindanao. Population attributable fraction (PAF) due to highest education attainment: 83.30% ([ratios of relative risks (RRR)], 9.69), 85.49% ([RRR], 10.69), and 81.96% ([RRR], 8.33), respectively; PAF due to low SES: 24.36% ([RRR], 1.97), 29.45% ([RRR], 1.98), and 53.22% ([RRR], 3.28), respectively; PAF due to adult functional illiteracy: 6.61% ([RRR], 2.16), 18.57% ([RRR], 2.53), and 26.71% ([RRR], 3.43), respectively. Our results indicated that the estimated risk of functional illiteracy attributable to poor sanitation facilities in the Visayas is 13.21% ([RRR], 1.86). Our results indicated that the estimated risk of functional illiteracy attributable to *P*. *vivax* infection, *T*. *trichiura* monoinfection, and moderate/high infection intensity class for *T*. *trichiura* in Mindanao were 0.53% ([RRR], 1.26), 4.20% ([RRR], 1.40), and 3.96% ([RRR], 1.82), respectively (Table 7). In Luzon and Mindanao, being female was negatively and significantly associated with the prevalence of functional illiteracy compared to being male (Table 6). Full results of the multinomial logistic regression models are presented in S3 Text.

**Table 6:** List of predictors considered as influencing the prevalence of functional illiteracy in school-aged children.

Predictors	Luzon		The Visayas		Mindanao	
	RRR ^a^ (95CI ^b^)	P-value	RRR ^a^ (95CI ^b^)	P-value	RRR ^a^ (95CI ^b^)	P-value
**Age in years (continuous)** ^c^	1.08 (0.85, 1.36)	0.55	1.18 (0.96, 1.46)	0.12	0.99 (0.72, 1.35)	0.95
**Female (versus Male)**	0.44 (0.24, 0.79)	0.01	0.70 (0.48, 1.02)	0.07	0.50 (0.28, 0.88)	0.02
**Below junior high school level (versus junior high school level completed)**	9.69 (3.27, 28.75)	*	10.69 (5.25, 21.77)	*	8.33 (3.53, 19.69)	*
**Mean functional literacy levels of the heads of household** **(adult functional literacy)** ^c^	2.16 (1.48, 3.14)	*	2.52 (1.97, 3.21)	*	3.43 (2.27, 5.17)	*
**Low SES (versus high SES)** ^d^	1.97 (1.52, 2.56)	*	1.98 (1.24, 3.16)	*	3.28 (1.65, 6.54)	*
**Main sources of drinking water for members of household: Lake/pond/rain water/well (versus Piped into dwelling)**	0.88 (0.48, 1.61)	0.67	0.72 (0.54, 0.94)	0.02	0.99 (0.50, 1.95)	0.98
**Types of toilet facility at home: No toilet/bush/field/pit toilet** **(versus Flush toilet)**	1.08 (0.50, 2.32)	0.85	1.86 (1.17, 2.93)	0.01	0.67 (0.28, 1.58)	0.36
**Main material of floor of houses: Sand/bamboo/palm/wood floor (versus Cement)**	1.60 (0.81, 3.14)	0.17	0.83 (0.71, 0.97)	0.02	1.64 (0.77, 3.51)	0.20
**Main material of outer walls of houses: Bamboo/palm/wood walls (versus Aluminium)**	0.95 (0.48, 1.90)	0.88	0.70 (0.45, 1.10)	0.12	1.41 (0.76, 2.63)	0.28
**Household education stimuli: Below the average total education stimuli scores (versus above the average total education stimuli scores)** ^c^	0.99 (0.76, 1.29)	0.94	1.15 (0.89, 1.48)	0.29	1.11 (0.73, 1.68)	0.63
**Prevalence of *P*. *falciparum*^҂^**	0.56 (0.33, 0.95)	0.03	N/A	N/A	0.75 (0.54, 1.04)	0.08
**Prevalence of *P*. *vivax*** ^c^	1.29 (0.81, 2.05)	0.28	N/A	N/A	1.26 (1.04, 1.52)	0.02
**Prevalence of *A*. *lumbricoides*** ^c^	1.10 (0.84, 1.46)	0.49	0.92 (0.71, 1.19)	0.51	-	-
**Prevalence of *T*. *trichiura*** ^c^	1.58 (0.72, 3.46)	0.26	0.88 (0.43, 1.77)	0.71	-	-
**Prevalence of *A*. *lumbricoides* monoinfection** ^c^	-	-	-	-	0.74 (0.44, 1.23)	0.24
**Prevalence of *T*. *trichiura* monoinfection** ^c^	-	-	-	-	1.40 (1.10, 1.78)	0.01
**Prevalence of *A*. *lumbricoides* and *T*. *trichiura* coinfection** ^c^	-	-	-	-	0.73 (0.52, 1.03)	0.07
**Intercept**	0.05 (0.02, 0.1)	*	0.05 (0.03, 0.08)	*	0.04 (0.02, 0.08)	*

Note: Reference group = functional literacy;

^a^ RRR = ratios of relative risks;

^b^ 95CI = 95% confidence interval;

^c^ Variables were standardized to have mean of zero, and standard deviation of one;

^d^ SES = socioeconomic status;

* Statistically significant (P<0.0001);

N/A = Not available.

**Table 7:** List of predictors considered as influencing the prevalence of functional illiteracy in school-aged children in Mindanao (infection intensity classes).

Predictors	RRR ^a^ (95CI ^b^)	P-value
**Age in years (continuous)** ^c^	1.03 (0.80, 1.33)	0.80
**Female (versus Male)**	0.48 (0.28, 0.83)	0.01
**Below Junior high school level (versus junior high school level completed)**	8.96 (4.03, 19.91)	*
**Mean functional literacy levels of the heads of household (adult functional literacy)** ^c^	3.80 (2.68, 5.40)	*
**Low SES (versus high SES)** ^d^	3.01 (1.58, 5.75)	*
**Main sources of drinking water: Lake/pond/rain water/well (versus piped into dwelling)**	0.97 (0.49, 1.93)	0.93
**Types of toilet facility at home: No toilet/bush/field/pit toilet (versus Flush toilet)**	0.58 (0.22, 1.51)	0.26
**Main material of floor of houses: Sand/bamboo/palm/wood floor (versus Cement)**	N/A	N/A
**Main material of outer walls of houses: Bamboo/palm/wood walls (versus Aluminium)**	0.97 (0.45, 2.11)	0.95
**Below the average total education stimuli scores (versus above the average total education stimuli scores)** ^c^	0.77 (0.59, 1.02)	0.07
**Prevalence of *P*. *falciparum*** ^c^	0.61 (0.43, 0.87)	0.01
**Prevalence of *P*. *vivax*** ^c^	1.53 (1.06, 2.23)	0.03
**Prevalence of low infection intensity class for *T*. *trichiura* infection** ^c^	0.93 (0.66, 1.32)	0.70
**Prevalence of moderate infection intensity class for *T*. *trichiura* infection** ^c^	1.82 (1.08, 3.09)	0.03
**Intercept**	0.04 (0.02, 0.10)	*

Note: Reference group = functional literacy;

^a^ RRR = ratios of relative risks;

^b^ 95CI = 95% confidence interval;

^c^ Variables were standardized to have mean of zero, and standard deviation of one;

^d^ SES = socioeconomic status;

* Statistically significant (P<0.0001);

N/A = Not available.

Across all regions, we observed reduced propensity of clustering and larger cluster sizes after adjusting for the effect of covariates (Table 8 and S6 Fig).

**Table 8:** Results of semivariograms for prevalence of functional illiteracy.

Functional illiteracy	Observed data	Model 1	Model 2	Model 3	Model 4	Model 5
**Luzon**						
**Partial sill**	0.0035	0.0013	0.0012	0.0013	0.0013	0.0010
**Nugget**	0.002	0.0003	0.0002	0.0005	0.0005	0.0004
**Practical range (km)** ^a^	0.0120 (1.33)	0.3042 (33.46)	0.1505 (16.55)	0.2401 (26.41)	0.2401 (26.41)	0.3013 (33.15)
**% of the variance due to cluster** ^b^	63.62	81.37	83.92	71.43	71.43	71.51
**The Visayas**						
**Partial sill**	0.0096	N/A	0.0117	0.0128	0.0134	0.0116
**Nugget**	0.0034	N/A	0.0061	0.0054	0.0067	0.0060
**Practical range (km)** ^a^	0.2770 (30.74)	N/A	1.4738 (162.12)	1.4542 (159.96)	1.4165 (155.81)	1.8647 (205.11)
**% of the variance due to cluster** ^b^	73.83	N/A	65.79	70.36	66.66	65.89
**Mindanao**						
**Partial sill**	0.0115	0.0111	0.0114	0.0122	0.0122	0.0113
**Nugget**	0.0126	0.0030	0.0031	0.0023	0.0024	0.0036
**Practical range (km)** ^a^	0.2290 (25.42)	0.3950 (43.45)	0.4019 (44.21)	0.3959 (43.55)	0.4005 (44.05)	0.4241 (46.65)
**% of the variance due to cluster** ^b^	47.66	78.53	78.88	84.27	83.84	75.80

Note:

^a^ Calculation based on practical range multiplied by 111, 1 decimal degree = 111km, 0.1 = 11km, 0.01 = 1km, 0.05 = 5km, 0.005 = 500m;

^b^ Calculation based on partial sill divided by sill (partial sill and nugget), multiplied by 100;

N/A = Not available.

**Fig 1:**
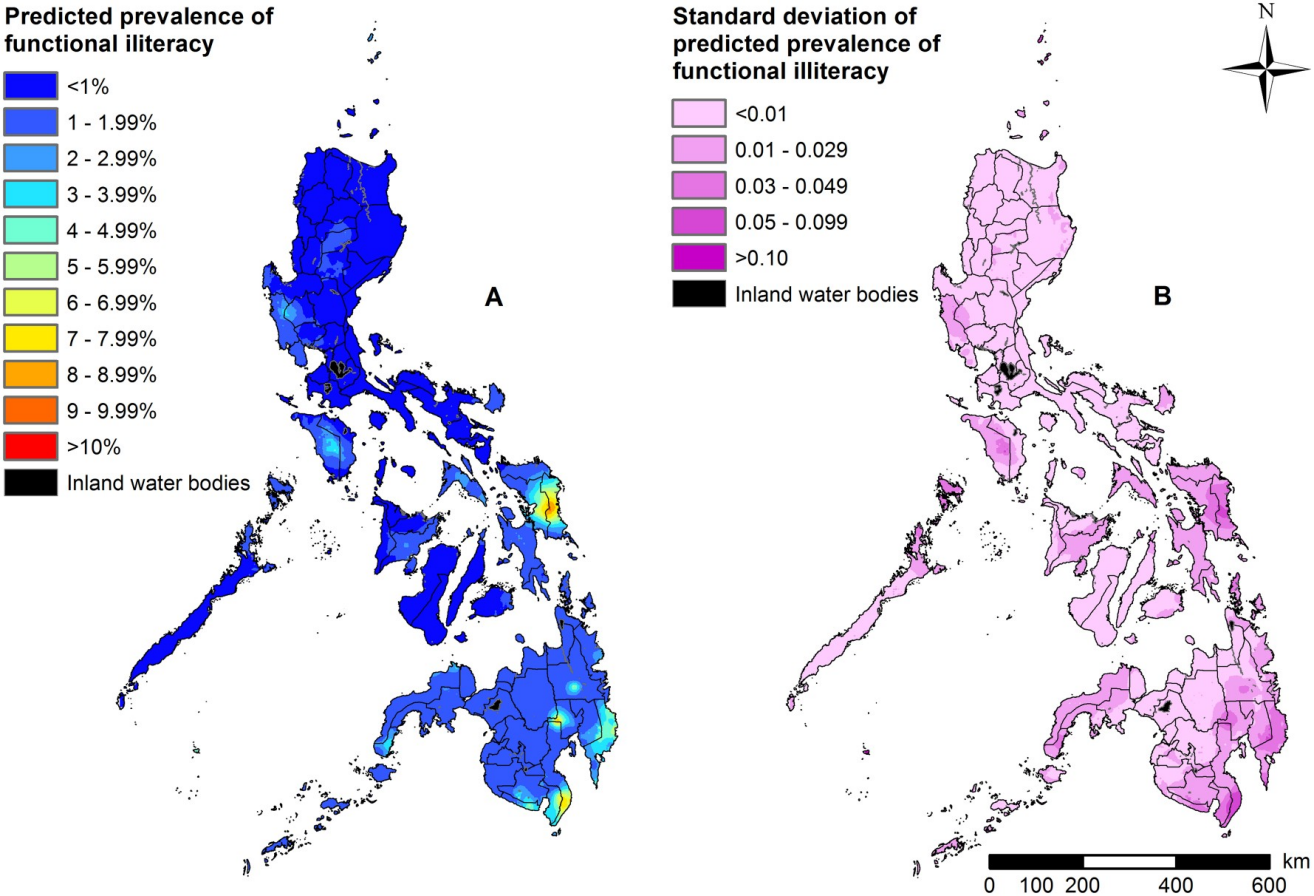
Maps of functional illiteracy in the Philippines. (A) Predicted prevalence. (B) Standard deviation of predicted prevalence. Note: Results based on Model 3 for Luzon and the Visayas; based on Model 5 for Mindanao.

Our predictive maps indicate significant spatial heterogeneity in the prevalence of each level of functional literacy within each region of the Philippines (Fig 1 and S7 Fig). Our predictive maps also demonstrate that the prevalence of functional illiteracy ranges between 1 and 3%, with the highest rates predicted in localized areas of the eastern region of the Visayas (Eastern Samar), and the centre (Davao Del Norte province) and the southwestern tip of Mindanao (Davao Occidental province) (up to 10.5%) (Fig 1).

The models showed reasonable discriminative ability for functional illiteracy (area under the curve [AUC] = 0.75 for Luzon, 0.75 for the Visayas, and 0.71 for Mindanao), low functional literacy (AUC = 0.70 for Luzon, and 0.74 for the Visayas), and moderate functional literacy (AUC = 0.74 for Luzon, 0.72 for the Visayas and 0.82 for Mindanao) (Table 9).

**Table 9:** Area under the receiver operating characteristic (ROC).

Region/ model	Threshold	Area under the ROC curve ^a^	Standard error	95% confidence interval
**Luzon**				
**Moderate Functional literacy**	20%	0.74	0.09	0.55, 0.92
**Low Functional literacy**	5%	0.70	0.07	0.57, 0.87
**Functional illiteracy**	3%	0.75	0.06	0.63, 0.88
**The Visayas**				
**Moderate Functional literacy**	20%	0.72	0.07	0.59, 0.85
**Low Functional literacy**	7%	0.74	0.07	0.59, 0.89
**Functional illiteracy**	7%	0.75	0.08	0.60, 0.90
**Mindanao**				
**Moderate Functional literacy**	20%	0.82	0.09	0.65, 0.98
**Low Functional literacy**	5%	0.66	0.07	0.52, 0.79
**Functional illiteracy**	6%	0.71	0.14	0.51, 0.90

Note:

^a^ ROC was used to determine discriminatory performance of the model predictions relative to observed mean prevalence of functional literacy as the cut-off value to determine discriminatory performance of the model predictions. An Area under the ROC curve of $0.50-0.6\bar{9}$ indicates a poor discriminative capacity, $0.70-0.8\bar{9}$ indicates a reasonable capacity, and ≥0.90 indicates a very good predictive performance.

For 2017, it was estimated that Luzon had the highest estimated number of school-aged individuals with functional illiteracy (estimated total 2,185), followed by Mindanao (estimated total 1,550) and the Visayas (estimated total 1,212) (Table 10).

**Table 10:** Predicted number of school-aged children (10 to 19 years old) in each functional literacy class in the Philippines in 2017.

Total population for 2015 (in Millions) ^a^	Annual population growth rate for 2015–2020 (Percent) ^a^	Individuals aged 10-19y (Percent) ^b^	Number of individuals aged 10–19 years ^c^			
			Functional Literacy Class	Regions		
				Luzon	The Visayas	Mindanao
100.7	1.48	19.7	**Moderate functional literacy**	127,177	44,658	42,467
			**Low functional literacy**	127,326	44,884	42,384
			**Functional illiteracy**	2,185	1,212	1,550

Note: The forecast for 2017 is based on a constant trend in the prevalence of selected functional literacy profiles.

^a^ Source: Alpha version 2015 estimates of numbers of people per grid square, with national totals adjusted to match UN population division estimates (http://esa.un.org/wpp/) and remaining unadjusted. SPATIAL RESOLUTION: 0.000833333 decimal degrees (approx. 100 m at the equator). PROJECTION: Geographic, WGS84. DATE OF PRODUCTION: November 2013 (195, 283);

^b^ Source: The World Population Prospects 2015 Revision Population Database (196, 197);

^c^ Estimated value based on the ArcGIS Map algebra raster calculator. 2017 population raster map was multiplied by the proportion of the Filipino population aged 10 to 19 years to derive a raster map of the number of school-aged individuals aged 10 to 19 years in 2017 (people per square kilometre). We then multiplied this raster map of the total population aged 10 to 19 years by our prediction maps of the prevalence of each of the categories of functional literacy (i.e. moderate functional literacy, low functional literacy, and functional illiteracy), adjusted for selected covariates in models in ArcGIS software (ESRI 2013. ArcGIS Desktop: Release 10. Redlands, CA: Environmental Systems Research Institute).

**Fig 2:**
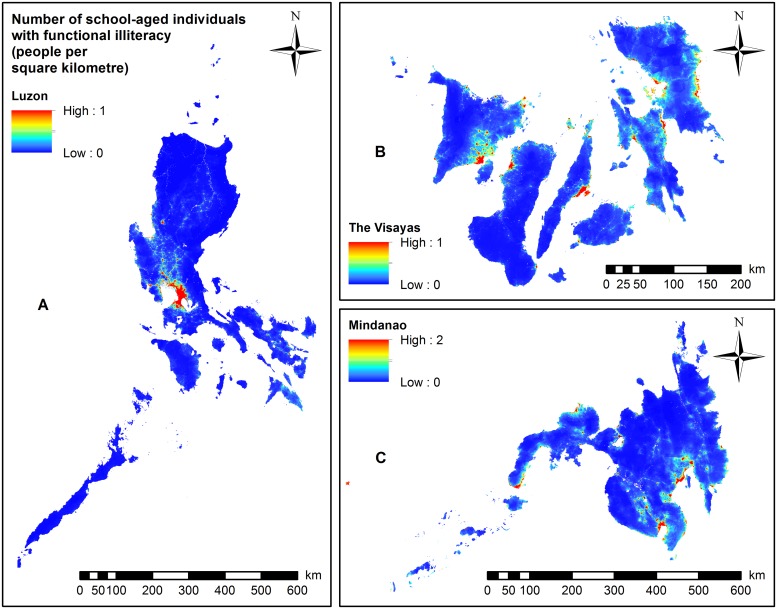
Maps showing the total number of school-aged individuals with functional illiteracy, 2017. (A) Luzon (Results based on Model 3). (B) The Visayas (Results based on Model 3). (C) Mindanao (Results based on Model 5).

**Fig 3:**
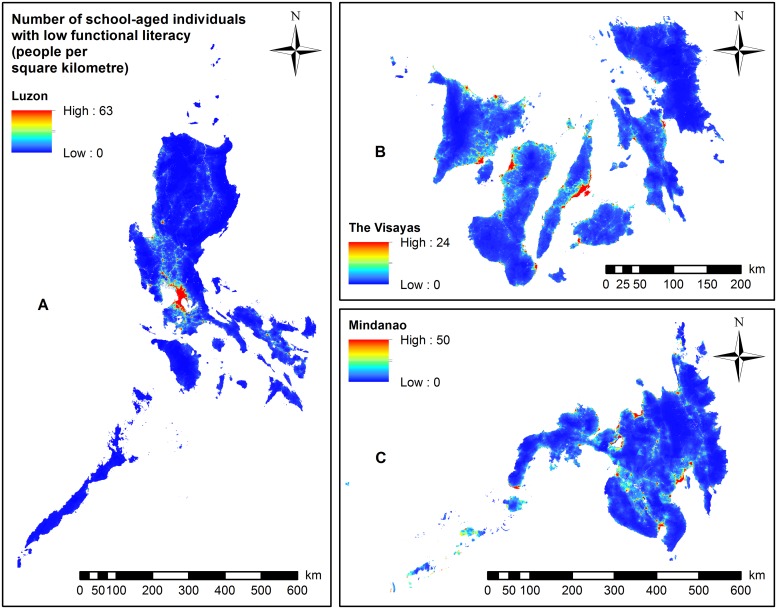
Maps showing the total number of school-aged individuals with low functional literacy, 2017. (A) Luzon (Results based on Model 3). (B) The Visayas (Results based on Model 3). (C) Mindanao (Results based on Model 5).

The highest number of school-aged individuals with functional illiteracy (Fig 2), low functional literacy (Fig 3), and moderate functional literacy (S8 Fig) was circumscribed to areas around metropolitan Manila, with some municipalities exceeding 63 people per square kilometre.

**Fig 4:**
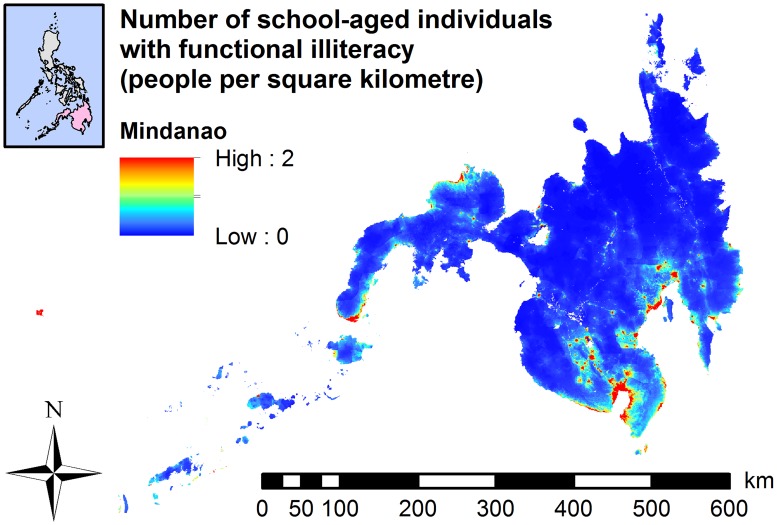
Map showing a total number of school-aged individuals with functional illiteracy, 2017, Mindanao, based on Model 6.

**Fig 5:**
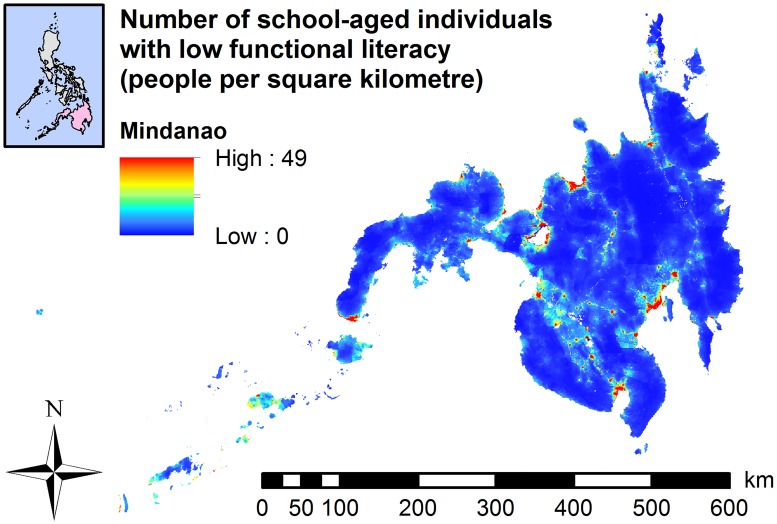
Map showing a total number of school-aged individuals with low functional literacy, 2017, Mindanao, based on Model 6.

In the Visayas (Fig 2) and Mindanao (Figs 2 and 4), the predicted number of school-aged individuals with functional illiteracy was widely distributed in a number of provinces. The highest numbers of school-aged individuals with moderate (S8 Fig) or low functional literacy (Fig 3) were predominantly localised in the western region of the Visayas (some municipalities with more than 24 persons per square kilometre). In Mindanao, the highest numbers of school-aged individuals with moderate (S8 and S9 Figs) or low functional literacy (Figs 3 and 5) were localised in the central and southern parts of Mindanao with some municipalities exceeding 50 people per square kilometre.

Names of provinces and municipalities with the poorest functional literacy indicators based on our analyses are provided in Table 11.

**Table 11:** Provinces and municipalities identified with the highest number of school-aged individuals with low levels of functional literacy.

Regions	Luzon	The Visayas	Mindanao
**Provinces (municipalities)**	The National Capital Region of the Philippines (Kalookan city, Malabon Manila, San Juan, Mandaluyong, and Valenzuela), Cavite (Dasmarinas, Bacoor), Laguna (San Pedro), Albay (Legazpi and Tabaco), Catanduanes (Virac), Sorsogon (Sorsogon), Camarines Sur (Naga), Camarines Norte (Daet), Benguet (Baguio), La Union (Agoo), Pangasinan (Dagupan), Tarlac (Tarlac), Nueva Ecija (Cabanatuan), Pampanga (Angeles, San Fernando, Mabalacat, Santo Tomas, and Macabebe), Zambales (Olongapo), Bataan (Dinalupihan, Orani, Samal, Balanga, and Limay), Bulacan (Calumpit, Hagonoy, Paombong, Pulilan, Baliuag, Plaridel, Malolos, Guiguinto, Balagtas, Bocaue, Marilao, San Jose Del Monte, and Meycauayan).	Eastern Samar (Borongan, San Julian, Sulat, and Dolores, Oras), Northern Samar (Catarman), Bohol (Tagbilaran), Cebu (Cordoba, Lapu-Lapu, Cebu, Mandaue, Consolacion, Talisay, Minglanilla, Bogo), Leyte (Isabel, Palompon, Bato, and Ormoc city), Southern Leyte (Maasin), Negros Occidental (Bacolod, Victorias), Negros Oriental (Dumaguete), Antique (San Jose), Iloilo (Iloilo city, Leganes, Pavia, Pototan, Santa Barbara, Oton, Estancia, Balasan, and Carles), Capiz (Roxas), Aklan (Kalibo).	Davao Oriental (Bagana, Caraga, Governor Generoso, Manay, and Mati), Davao Del Norte (Talaingod and Santo Tomas), Davao Occidental (Malita, Don Marcelino, Jose Abad Santos, and Sarangani island), Lanao Del Sur (Marawi, Piagapo, Taraka, and Tamparan), Maguindanao (Cotabato), Misamis Oriental (Cagayan De Oro, Tagoloan, Plaridel, Oroquieta, Jimenez, and Ozamis), Sarangani (Alabel, Glan, Malapatan, Malungon, Kiamba, and Maasim), South Cotabato (General Santos, T’ Boli, and Lake Sebu), Tawi-Tawi island which is an island province located in the Autonomous Region in Muslim Mindanao (Mapun), Zamboanga Del Sur (Molave, Pagadian, and Zamboanga), Zamboanga Del Norte (Dapital and Dipolog), and Zamboanga Sibugay (Ipil).

## Discussion

Overall, our findings suggest significant spatial heterogeneity in the prevalence of functional literacy indicators within each region of the Philippines, reflecting variability in the determinants and the need for location-specific interventions.

Our findings are consistent with previous studies, indicating that multiple factors exert a negative impact on functional literacy in school-aged children. Evidence suggests a possible link between low socioeconomic status and cognitive function, including children’s levels of language and literacy skills [17]. This relationship is mediated by different mechanisms such as parenting behaviour and household linguistic stimulation [17]. The observed association could also be explained by the effect of malnutrition in the poorest areas of the Philippines [27].

We found that the determinants of functional literacy are region-specific. For example, in Luzon and Mindanao, our results indicate that female school-aged children are at less risk of functional illiteracy compared to males, suggesting that girls may be less exposed to factors that affect their cognitive development compared to boys. The gender difference identified in our study could also be partly explained by integrated helminth control programs, which provide more attention to female adolescents [28], and the fact that boys are often involved with agricultural activities in resource-limited areas, limiting their participation in schooling or academic learning as agriculture in the Philippines has been dominated by males [29].

In contrast, our results for the Visayas showed that higher prevalence of functional illiteracy was associated with households with poor sanitation facilities. School-aged children who live in socioeconomically deprived environments face multiple challenges such as non-utilisation of sanitary facilities, open defecation, and limited education services. Previous studies have demonstrated that open defecation, a practice highly prevalent in the Visayas [11], is associated with high prevalence of STH infections [30]. Deficient sanitation promotes not only the transmission of STH infections, but also water-borne infections and diarrhoea. It also increases the associated risks of malabsorption, malnutrition, and iron-deficiency anaemia, which could aggravate cognitive dysfunction [31].

Our results also showed that the utilisation of unprotected water sources such as wells and lakes as main sources of drinking water at home is negatively associated with the prevalence of functional illiteracy in the Visayas. Our findings may be confounded by the fact that unprotected drinking water sources are more likely to be present in agricultural communities where access to food and nutritional security are assured through local food production [32]. Further investigation is needed to examine the factors mediating the relation between access to water sources and the prevalence of functional illiteracy identified in this study.

The risk of STH-associated morbidity depends on the intensity of STH infection and the species of STH [33]. This study demonstrated significant geographical variation in the burden of functional illiteracy in school-aged children, which could possibly be explained by *T*. *trichiura* infection. Indeed, our findings suggest the risk of functional illiteracy among school-aged children in Mindanao might be reduced by 4.20% by preventing *T*. *trichiura* infection. These results could be explained by the pathophysiological impact of *T*. *trichiura* infection, including chronic diarrhoea, malnutrition, and iron-deficiency anaemia, all of which are associated with impaired cognitive function [34]. Furthermore, a recent experimental study demonstrated that *T*. *trichiura* contributes to pathological changes in the hippocampus and amygdala [10]. Existing preventive chemotherapy shows low to moderate efficacy against *T*. *trichiura* in high endemic countries [35]. This finding suggests that new solutions such as alternative treatment (e.g. oxantel pamoate) are needed to eliminate STH-associated morbidity.

Our results show that large areas in the Philippines still lag in meeting functional literacy targets. Our estimates indicated that for 2017, Luzon had the highest estimated number of school-aged individuals with low levels of functional literacy. However, when these estimates were adjusted by the geographical variation in population density we found that areas in Mindanao had the highest density of school-aged individuals with functional illiteracy. Geographically targeted functional literacy interventions should thus be prioritised in at-risk areas identified in each region. These interventions should consider the region-specific determinants highlighted above. For example, in Luzon, the gender difference in the prevalence of reduced functional literacy identified in our study suggests that the current integrated helminth control program, which recently expanded its target age group from 1 to 12 years old to individuals 1 to 18 years old [28], should provide more attention to male adolescents. In the Visayas, given the high attributable risk of functional illiteracy from poor sanitation facilities, health educational programs promoting appropriate hygiene and sanitation practice such as educational videos (e.g. The Magic Glasses), which have proven efficacy in China [36], and are currently being tested in the Philippines are recommended. In Mindanao, given the perceived risk of functional illiteracy that could be associated with *T*. *trichiura* infections, and the chemotherapeutic failure for this particular parasite, educational and health promotion programs to this region should be considered.

This study has some limitations that need consideration. Our data are from the 2008 Functional Literacy, Education and Mass Media Survey (FLEMMS) and may not accurately reflect the current situation. That said, these data constitute the best available and most contemporaneous dataset available and represents a cross section of the school-age population in the Philippines. Additionally, the rate of functional literacy has not seen much improvement in the last three FLEMMS (83.8% in 1994, 84.1% in 2003, and 86.4% in 2008) suggesting that data from the 2008 survey were unlikely to differ notably from the current situation. The prediction figures for 2017 may have overestimated the numbers of children currently at risk of functional illiteracy because this model assumes a constant increase in population and it does not account for changes in the prevalence of risk factors of functional illiteracy and STH infection such as poverty and WASH, with the rapid urbanisation that is happening in some parts of the Philippines [37]. However, according to the Philippines Statistics Authority (PSA), while the Philippines has shown some progress achieving the SDGs for literacy, poverty, and water sanitation, the levels have fluctuated between each survey period, showing no distinct trend, however there remain some parts of the Philippines, such as Autonomous Region in Muslim Mindanao (ARMM), that constantly record above average rates of poverty and less access to safe drinking water and sanitation facilities [38, 39]. Presently, there are no data available on functional literacy in the Philippines for 2017 to validate our prediction results, therefore further investigation is required.

In addition, our predictive prevalence for *P*. *falciparum* and *P*. *vivax* are likely to represent underestimates, as malaria data for the targeted age group of our study were not available. However, given the low endemicity level of both species of malaria across the Philippines, the effect of malaria was subtle. Small-scale factors such as nutrition are also known to be important determinants of cognitive dysfunction [6]. Unfortunately, data on these factors were not available. In addition, our estimated PAF could be biased in the presence of unaccounted confounding factors.

In conclusion, our findings support the need for spatially targeted strategies that can lead to a reduction in the transmission of STH infections and other determinants of functional illiteracy in school-aged children in the Philippines. In the context of the current work, this is particularly relevant in order for the Philippines to achieve the SDG target for functional literacy by 2030.

## Supporting information

S1 Text Description of FLEMMS survey. (PDF)

S2 Text Description of indicators. (PDF)

S3 Text Multinomial logistic regression models and preliminary analysis. (PDF)

S4 Text Estimation of the population attributable fraction (PAF). (PDF)

S5 Text Model specification. (PDF)

S6 Text Prediction maps and standard deviation maps. (PDF)

S7 Text Estimation of the number of school-aged children in each functional literacy class in the Philippines in 2017. (PDF)

S1 Fig Map of 2008 FLEMMS survey locations. Note: Figure produced by authors of this paper and previously published in Int J Environ Res Public Health [19] and reused under CC BY license.
(PDF)

S2 Fig Maps showing predicted prevalence of malaria infections. (A) *Plasmodium falciparum*. (B) *Plasmodium vivax*. Note: *Pf*PR_2-10_ = *P*. *falciparum* parasite rate in the 2 to 10 years; *Pv*PR_2-10_ = *P*. *vivax* parasite rate in the 2 to 10 years; Areas with no colours indicate predominantly *P*. *falciparum* and *P*. *vivax* free areas [20, 40].
(PDF)

S3 Fig Maps showing predicted prevalence of STH infections. (A) *A*. *lumbricoides*. (B) *T*. *trichiura*. (C) Hookworm. Note: Figure produced by authors of this paper and previously published in PLoS Negl Trop Dis [12] and reused under CC BY license.
(PDF)

S4 Fig Maps showing predicted prevalence of mono- and co-infection. (A) *A*. *lumbricoides* monoinfection. (B) *T*. *trichiura* monoinfection. (C) *A*. *lumbricoides* and *T*. *trichiura* co-infection. Note: Figure produced by authors of this paper and previously published in Parasit and Vectors [21] and reused under CC BY license.
(PDF)

S5 Fig Bar graph showing basic household WASH characteristics in the Philippines. Note: A higher number of households in Mindanao were accessing unprotected wells (11.7% in Mindanao compared to 5% in Luzon and 7% in the Visayas), and had access to either closed-pit or open-pit latrines compared to the Visayas and Luzon (18% in Mindanao compared to 6.7%, 5.2%, respectively). A higher proportion of households in the Visayas were practising open-defecation compared to Luzon and Mindanao (12.5%, 4.7% and 6.4%, respectively). Figure produced by authors of this paper and previously published in Int J Environ Res Public Health [19] and reused under CC BY license.
(PDF)

S6 Fig Semivariograms of prevalence of functional illiteracy. Note: Semivariograms of prevalence of observed functional illiteracy indicators and residuals for the final multinomial models (residual semivariograms) at each region to examine the presence of spatial autocorrelation (in decimal degrees). Above semivariograms indicated residual spatial dependency in the prevalence of moderate and low functional literacy indicators in Luzon. In the Visayas, after adjusting for the covariates, residual spatial dependency of functional illiteracy was no longer evident in the semivariograms. In Mindanao, our results indicated residual spatial dependency in the prevalence of moderate functional literacy and functional illiteracy.
(PDF)

S7 Fig Maps of predicted prevalence of functional literacy indicator classes. (A) Moderate functional literacy. (B) Low functional literacy. Note: Results based on Model 3 for Luzon and the Visayas; based on Model 5 for Mindanao.
(PDF)

S8 Fig Maps showing the total number of school-aged individuals with moderate functional literacy, in the Philippines by region (2017). (A) Luzon. (B) The Visayas. (C) Mindanao. Note: Results based on Model 3 for Luzon and the Visayas; based on Model 5 for Mindanao.
(PDF)

S9 Fig Map showing the total number of school-aged individuals with moderate functional literacy, 2017, Mindanao, based on infection intensity classes for *T*. *trichiura*. (PDF)

S1 Table Table showing the scoring of household education stimuli (home-inventory proxy) indicators. (PDF)

S2 Table Table showing demographic characteristics of the heads of households, stratified by regions of the Philippines. (PDF)

S3 Table Table showing socioeconomic status of households, stratified by regions of the Philippines. (PDF)

S4 Table Table showing household education stimuli score, stratified by regions of the Philippines. (PDF)
