# Correction: Functional illiteracy burden in soil-transmitted helminth (STH) endemic regions of the Philippines: An ecological study and geographical prediction for 2017

**DOI:** 10.1371/journal.pntd.0010553

**Published:** 2022-06-16

**Authors:** The PLOS Neglected Tropical Diseases Staff

In Table 6, the row starting with “Prevalence of *P*. *falciparum*” is incorrectly marked with a ҂ symbol. This should instead be a superscript letter ‘c’, as on the adjacent rows.

Please see the corrected Table 6 below.

**Table 6:** List of predictors considered as influencing the prevalence of functional illiteracy in school-aged children.

Predictors	Luzon		The Visayas		Mindanao	
	RRR ^a^ (95CI ^b^)	P-value	RRR ^a^ (95CI ^b^)	P-value	RRR ^a^ (95CI ^b^)	P-value
**Age in years (continuous)** ^c^	1.08 (0.85, 1.36)	0.55	1.18 (0.96, 1.46)	0.12	0.99 (0.72, 1.35)	0.95
**Female (versus Male)**	0.44 (0.24, 0.79)	0.01	0.70 (0.48, 1.02)	0.07	0.50 (0.28, 0.88)	0.02
**Below junior high school level (versus junior high school level completed)**	9.69 (3.27, 28.75)	*	10.69 (5.25, 21.77)	*	8.33 (3.53, 19.69)	*
**Mean functional literacy levels of the heads of household (adult functional literacy)** ^c^	2.16 (1.48, 3.14)	*	2.52 (1.97, 3.21)	*	3.43 (2.27, 5.17)	*
**Low SES (versus high SES)** ^d^	1.97 (1.52, 2.56)	*	1.98 (1.24, 3.16)	*	3.28 (1.65, 6.54)	*
**Main sources of drinking water for members of household: Lake/pond/rain water/well (versus Piped into dwelling)**	0.88 (0.48, 1.61)	0.67	0.72 (0.54, 0.94)	0.02	0.99 (0.50, 1.95)	0.98
**Types of toilet facility at home: No toilet/bush/field/pit toilet (versus Flush toilet)**	1.08 (0.50, 2.32)	0.85	1.86 (1.17, 2.93)	0.01	0.67 (0.28, 1.58)	0.36
**Main material of floor of houses: Sand/bamboo/palm/wood floor (versus Cement)**	1.60 (0.81, 3.14)	0.17	0.83 (0.71, 0.97)	0.02	1.64 (0.77, 3.51)	0.20
**Main material of outer walls of houses: Bamboo/palm/wood walls (versus Aluminium)**	0.95 (0.48, 1.90)	0.88	0.70 (0.45, 1.10)	0.12	1.41 (0.76, 2.63)	0.28
**Household educational stimuli: Below the average total education stimuli scores (versus above the average total education stimuli scores)** ^c^	0.99 (0.76, 1.29)	0.94	1.15 (0.89, 1.48)	0.29	1.11 (0.73, 1.68)	0.63
**Prevalence of *P*. *falciparum*** ^c^	0.56 (0.33, 0.95)	0.03	N/A	N/A	0.75 (0.54, 1.04)	0.08
**Prevalence of *P*. *vivax*** ^c^	1.29 (0.81, 2.05)	0.28	N/A	N/A	1.26 (1.04, 1.52)	0.02
**Prevalence of *A*. *lumbricoides*** ^c^	1.10 (0.84, 1.46)	0.49	0.92 (0.71, 1.19)	0.51	-	-
**Prevalence of *T*. *trichiura*** ^c^	1.58 (0.72, 3.46)	0.26	0.88 (0.43, 1.77)	0.71	-	-
**Prevalence of *A*. *lumbricoides* monoinfection** ^c^	-	-	-	-	0.74 (0.44, 1.23)	0.24
**Prevalence of *T*. *trichiura* monoinfection** ^c^	-	-	-	-	1.40 (1.10, 1.78)	0.01
**Prevalence of *A*. *lumbricoides* and *T*. *trichiura* coinfection** ^c^	-	-	-	-	0.73 (0.52, 1.03)	0.07
**Intercept**	0.05 (0.02, 0.1)	*	0.05 (0.03, 0.08)	*	0.04 (0.02, 0.08)	*

Note: Reference group = functional literacy;

^a^ RRR = ratios of relative risks;

^b^ 95CI = 95% confidence interval;

^c^ Variables were standardized to have mean of zero, and standard deviation of one;

^d^ SES = socioeconomic status;

* Statistically significant (P<0.0001);

N/A = Not available.
