# Supplementary material for: Functional illiteracy burden in soil-transmitted helminth (STH) endemic regions of the Philippines: An ecological study and geographical prediction for 2017
Source: PLoS Negl Trop Dis. 2019 Jun 21;13(6):e0007494. doi: 10.1371/journal.pntd.0007494 (PMC6588226; doi:10.1371/journal.pntd.0007494)
Supplement: S1 Text — (PDF) [file pntd.0007494.s001.pdf]

S1 Text. Description of FLEMMS survey

Sampling methods of FLEMMS

Functional literacy data using in this study were collected during the 2008 Functional Literacy, Education and Mass Media Survey (FLEMMS) [1]. Primary sampling units (PSUs) within a region were stratified based on the proportion of households in the barangay engaged in agricultural activities and per capita income of the city/municipality. In the first stage, PSU were selected with probability proportional to the number of households in the 2000 CPH. PSUs consisted of a barangay or a group of contiguous barangays. In the second stage, in each sample PSU, EAs were selected with probability proportional to the number of households in 2000 CPH. An EA is defined as an area with discernible boundaries consisting of approximately 350 contiguous households. In the third stage, from each sampled EA, housing units were selected using systematic sampling. For operational considerations, at most 30 housing units were selected per sample. All sample households were interviewed from November 20, 2008 to December 13, 2008 [1]. According to the FLEMMS report, the response rate for the 2008 FLEMMS survey was 94.7% and 87.5% for the household survey and the individual survey respectively.

The original 2008 FLEMMS functional literacy classifications

According to 2008 FLEMMS, functionally literacy are defined as "those who can at least read, write, compute and/or comprehend, this also classifies persons who graduated from high school or completed higher level of education as functional literate". In the original FLEMMS functional literacy levels were stratified into 4 classifications – those who cannot read and write are classified as functional illiterate; those who can read and write (who can read and write with understanding a simple message in any language or dialect) are considered as basic literate or has simple literacy; those who can read, write, compute and comprehend (with a significantly higher level of literacy which includes not only reading and writing skills but also numerical and comprehension skills) are considered as functional literate. According to 2008 FLEMMS, participants who are classified as high school students were also considered as functional literate.

According to the original 2008 FLEMMS classification, participants who were classified as high school students were automatically considered as functionally literate. However, the authors considered that this decision introduced undue ambiguity in the definition of functional literacy (i.e. being high school students may not necessarily imply that they are functionally literate). For this reason, we excluded those who are classified in this category (i.e. approximately 17% of participants were excluded due to this reason).

The 2008 Functional Literacy, Education and Mass Media Survey (FLEMMS)

The 2008 FLEMMS is the fourth in a series of functional literacy surveys conducted in the Philippines. The previous three rounds were conducted in 1989, 1994 and 2003, respectively. The 2008 FLEMMS survey used the 2003 survey master sample (MS) created for household surveys on the basis of the 2000 Census Population and Housing (CPH) results [1]. In brief, for each region, a three-stage sampling scheme was used: the selection of primary sampling units (PSU) for the first stage, of sample enumeration areas (EA) for the second stage, and of sample housing units for the third stage. The heads of household were administered with the household questionnaire, and all information was gathered by trained interviewers (a copy of the FLEMMS forms available for reference from corresponding author). All members 10 to 64 years old in the sample households, regardless of educational attainment, were provided with a self-administered individual questionnaire (prepared in English and translated into 26 local languages commonly spoken in the selected sample areas).

References

1. The Philippines National Statistics Office. 2008 FLEMMS Final Report [Technical Report]. Manila: The Philippines National Statistics Office; 2008 [cited 2015 February 19]. Available from: https://psa.gov.ph/sites/default/files/2008%20FLEMMS%20FINAL%20REPORT.pdf.
