# Supplementary material for: Functional illiteracy burden in soil-transmitted helminth (STH) endemic regions of the Philippines: An ecological study and geographical prediction for 2017
Source: PLoS Negl Trop Dis. 2019 Jun 21;13(6):e0007494. doi: 10.1371/journal.pntd.0007494 (PMC6588226; doi:10.1371/journal.pntd.0007494)
Supplement: S2 Text — (PDF) [file pntd.0007494.s002.pdf]

S2 Text. Description of indicators

Functional literacy indicators

Functional literacy was estimated based on seven themes/questions included in the 2008 Functional Literacy, Education and Mass Media Survey (FLEMMS) individual questionnaire, using value set of 'satisfactory', and 'not satisfactory' (which also includes 'not answered independently' or 'no answer').

Sociodemographic indicators

For the purpose of our analysis, school-aged children were defined as 10 to 19 years old (school-aged children and adolescents). This age range has been chosen since the age for enrolment into secondary school may vary [1]. We used data from the FLEMMS individual questionnaires and FLEMMS household questionnaires on age, sex, education attainment level (completed no schooling, elementary level, or higher), marital status (single, married, divorced or widow), adult functional literacy (of head of households), and employment status (employed or unemployed).

Water, sanitation and hygiene (WASH) indicators

We used data from the FLEMMS household questionnaire on main sources of drinking water, the types of toilet facility at home, main material of floor, main material of roof, and main material of outer walls of houses. Each of the individual WASH items were categorized into binary variables: a) main sources of drinking water (piped into dwelling or other water source; e.g. well, lake, pond, or rain water), b) the types of toilet facility at home (flush toilet or other toilet types; e.g. pit toilet, no toilet or bush), c) main material of floor (cement or other material; e.g. sand, bamboo, palm or wood), d) main material of roof (aluminium or other material; e.g. bamboo, palm or wood), and e) main material of outer walls of houses (cement or other material; e.g. bamboo, cane, palm or wood).

Socioeconomic status (SES) indicators

We used data from the FLEMMS household questionnaire which included a poverty indicator (dichotomous variable: poor or non-poor) generated using ownership of household amenities and conveniences (e.g. whether the home had electricity, refrigerator, washing machine, phone, cell phones, TV, CD, Karaoke machine, personal computer, tractor, boat, car, tricycle, bicycle) [2]. We used this binary poverty indicator as a proxy of household-level SES in order to classify households as either low or high SES.

Household education stimuli indicators

A total of 19 close-ended questions were selected from FLEMMS household questionnaire as the home inventory-proxy items. These included questions such as 'Does your family read newspapers?', 'Does your family listen to radio?', and 'Is there a personal computer at home?' The items were used to construct cognitive stimulation sub-indices. All of the individual items were translated into dichotomous (yes or no) variables. The total score is the summation of the individual item scores and was used as a covariate in our models. The HOME (Home Observation for Measurement of the Environment) inventory has been used globally and in a wide variety of studies to measure the quality of cognitive stimulation and emotional support provided by a child's parents and family [3-6]. Because there were no formal HOME inventory assessments conducted during FLEMMS, we constructed our education stimuli measure using selected variables in the FLEMMS household questionnaire that are part of the HOME, following a procedure described elsewhere [7]. This is consistent with the scoring format of the Early Adolescent HOME inventory version for children aged 10 to 15 years old which contains 60 items clustered into 7 sub-index: 1) physical environment, 2) learning materials, 3) modelling, 4) instructional activities, 5) regulatory activities, 6) variety of experience, and 7) acceptance and responsivity, using a binary-choice (yes/no) format in scoring items for the HOME [4, 5].

Geographical distribution of STH infection data

We only considered data for *A. lumbricoides* and *T. trichiura* because coinfections with these parasites were most prevalent. We analysed infection intensity data only from Mindanao because the majority of STH infections for Luzon and the Visayas were of light infection intensity. The dataset was organised to estimate the prevalence of infection intensity classes as defined by WHO [8]. According to WHO definition, infection intensity classes are categorised according to eggs per gram of faeces (epg), into no-infection (0 epg); light-intensity infection; moderate-intensity infection and high-intensity infection. For *A. lumbricoides* infections, it is categorised into 0; 1-4,999; 5,000-49,999 epg, and over 50,000, respectively. For *T. trichiura* infections, it is categorised into 0, 1-999 epg, 1,000-9,999 epg, and over 10,000 epg, respectively. We combined moderate and high infection intensity classes due to low prevalence of high infection intensity classes for all species of STH in our analyses. We used predictive maps of soil-transmitted helminth prevalence generated from spatial analysis of the data collected during the most recent 2005 to 2007 National Schistosomiasis Survey in the Philippines [9-11]. Maps of predicted prevalence of STH infections, *A. lumbricoides* and *T. trichiura* monoinfection, coinfections and infection intensity classes for *A. lumbricoides* and *T. trichiura* used in our models were developed using Bayesian geostatistical models of STH prevalence including age and sex of individuals,

environmental variables (rainfall, land surface temperature and distance to inland water bodies) as predictors [12]. We extracted predicted values of STH infection profiles for each FLEMM survey location in ArcGIS version 10.4.0.5524 [13].

Geographical distribution of *P. falciparum* and *P. vivax* parasite rate

Spatial predicted values of *P. falciparum* (*Pf*PR_{2-10}) and *P. vivax* (*Pv*PR_{2-10}) parasite rate for children 2 to 10 years of age were created by the Malaria Atlas Project using model-based geostatistical models [14]. We extracted these predicted values of malaria endemicity for each FLEMM survey location in ArcGIS version 10.4.0.5524 [13].

References

1. United Nations Educational Scientific and Cultural Organisation. International standard classification of education: ISCED 1997 [Technical Report]. Paris: UNESCO; 2006 [cited 2015 December 14]. Available from: http://www.unesco.org/education/information/nfsunesco/doc/isced_1997.htm.
2. The Philippines National Statistics Office. 2008 FLEMMS Final Report [Technical Report]. Manila: The Philippines National Statistics Office; 2008 [cited 2015 February 19]. Available from: https://psa.gov.ph/sites/default/files/2008%20FLEMMS%20FINAL%20REPORT.pdf.
3. Bradley RH. Children's home environments, health, behavior, and intervention efforts: a review using the HOME inventory as a marker measure. Genet Soc Gen Psychol Monogr. 1993;119(4):437-90.
4. Bradley RH. Constructing and Adapting Causal and Formative Measures of Family Settings: The HOME Inventory as Illustration. J Fam Theory Rev. 2015;7(4):381-414.
5. Bradley RH, Caldwell BM. The relation of infants' home environments to achievement test performance in first grade: a follow-up study. Child Dev. 1984;55(3):803-9.
6. Frankenburg WK, Coons CE. Home Screening Questionnaire: its validity in assessing home environment. J Pediatr. 1986;108(4):624-6.
7. The U.S Bureau of Labor Statistics. NLSY79 - HOME scales and items: children 10 to 14 years old: National longitudinal survey of youth, children and young adults [Document on the Internet]. Washington, DC: The U.S Bureau of Labor Statistics; 1979 [cited 2016 December 14]. Available from: https://www.nlsinfo.org/content/cohorts/nlsy79-children/topical-guide/assessments/home-home-observation-measurement.
8. World Health Organization. Prevention and control of schistosomiasis and soil-transmitted helminthiasis [Technical Report]. Geneva: World Health Organization; 2002 [cited 2015 April 16]. Available from: http://www.who.int/intestinal_worms/resources/who_trs_912/en/.
9. Leonardo LR, Rivera P, Saniel O, Villacorte E, Crisostomo B, Hernandez L, et al. Prevalence survey of schistosomiasis in Mindanao and the Visayas, The Philippines. Parasitol Int. 2008;57(3):246-51.
10. Tarafder MR, Balolong E, Jr., Carabin H, Belisle P, Tallo V, Joseph L, et al. A cross-sectional study of the prevalence of intensity of infection with Schistosoma japonicum in 50 irrigated and rain-fed villages in Samar Province, the Philippines. BMC Public Health. 2006;6:61.
11. Soares Magalhães RJ, Salamat MS, Leonardo L, Gray DJ, Carabin H, Halton K, et al. Mapping the Risk of Soil-Transmitted Helminthic Infections in the Philippines. PLoS Negl Trop Dis. 2015;9(9):e0003915.
12. Owada K, Lau CL, Leonardo L, Clements ACA, Yakob L, Nielsen M, et al. Spatial distribution and populations at risk of A. lumbricoides and T. trichiura co-infections and infection intensity classes: an ecological study. Parasit Vectors. 2018;11(1):535.
13. Environmental Systems Research Institute. ArcGIS 10.4 for Desktop [Document on the Internet]. Redlands, CA: Environmental Systems Research Institute; 2015 [cited 2016 January 13]. Available from: http://www.esri.com/.
14. Gething PW, Patil AP, Smith DL, Guerra CA, Elyazar IR, Johnston GL, et al. A new world malaria map: Plasmodium falciparum endemicity in 2010. Malar J. 2011;10:378.
