# Supplementary material for: Functional illiteracy burden in soil-transmitted helminth (STH) endemic regions of the Philippines: An ecological study and geographical prediction for 2017
Source: PLoS Negl Trop Dis. 2019 Jun 21;13(6):e0007494. doi: 10.1371/journal.pntd.0007494 (PMC6588226; doi:10.1371/journal.pntd.0007494)
Supplement: S3 Text — (PDF) [file pntd.0007494.s003.pdf]

S3 Text. Multinomial logistic regression models and preliminary analysis

In Model 1 we included the sociodemographic, SES, WASH, and household education stimuli variables, in addition to predicted prevalence of *P. vivax* and *P. falciparum*; in Model 2 we included variables in Model 1 plus predicted prevalence of *A. lumbricoides*; in Model 3 we included variables in Model 2 plus predicted prevalence of *T. trichiura;* in Model 4 we included variables in Model 3 plus predicted prevalence of hookworm; in Model 5 we included variables in Model 1 and predicted prevalence of *A. lumbricoides* and *T. trichiura* monoinfection, and coinfections. STH coinfections with hookworms were not examined due to low prevalence. In all models, survey data were aggregated into groups according to sociodemographic indicators, SES, WASH, household education stimuli, predicted prevalence of infections, and location. All continuous predictor variables were standardized to have mean of zero, and standard deviation of one. These include age, adult functional literacy rate, household education stimuli scores, and infection data. Additionally, sex, marital status, WASH, SES, highest education, employment status at the time of the survey were categorized into binary variables (yes or no).

Our preliminary analysis showed that the prevalence of *T. trichiura* monoinfection was positively and significantly associated with the prevalence of functional illiteracy in Mindanao, thus we also developed a model which included sociodemographic, SES, WASH, household education stimuli, predicted prevalence of *P. vivax, P. falciparum*, and *T. trichiura* infection intensity classes (Model 6). This was done to allow estimation of the effect size of infection intensity classes for *T. trichiura* for this region. Infection intensity classes were only tested for Mindanao region due to unavailability of maps of predicted prevalence of infection intensity classes for Luzon and the Visayas (Owada et al, unpublished). Collinearity between covariates was measured using pairwise correlation coefficients, estimated in Stata version 13.1 [1]. Covariates were included in the final model based on backwards-stepwise regression analysis (with Wald's $P>0.2$ as the exclusion criterion, and $P< 0.05$ as the entry criterion). An interaction between age and sex was checked in all models using the "*mfpigen*" command in Stata version 13.1 [1].

The average age of school-aged children was 13.6 years. Highest education attainment was very similar across all three regions, with more than half of our samples completed up to primary level education. However, Mindanao had the highest number of school-aged children who had not completed any grade. A higher proportion of households was classified as low SES in Mindanao compared to Visayas and Luzon (49.9%, 42.6% and 33.2%, respectively; $P<0.05$). Luzon had higher average total education stimuli scores compared to the Visayas and Mindanao (8.12, 7.56, 7.03, respectively; $P<0.05$). In our preliminary analyses, marital status and employment status of heads of households were not found to be significantly associated with the prevalence of functional literacy in the multivariable models, thus these covariates were excluded from further analysis (Wald's $P>0.2$). We found collinearity between functional literacy and education attainment of heads of households, which provided support only for the inclusion of adult functional literacy as a predictor variable in our model.

In all three regions, highest education attainment completed, SES and adult functional illiteracy were positively and significantly associated with the prevalence of functional illiteracy (Table 6 in main manuscript). Households that utilised natural water sources such as lakes, ponds and wells as their main source of drinking water at home were negatively and significantly associated with the prevalence of functional illiteracy in the Visayas. Additionally, households that used pit toilets or had no access to toilet facilities (i.e. bush and field) were positively and significantly associated with the prevalence of functional illiteracy in the Visayas.

After accounting for the effects of other covariates our results on the role of STH and malaria infections indicated that in Luzon, prevalence of *P. falciparaum* infection was negatively and significantly associated with prevalence of low functional literacy, and functional illiteracy (Table 6 in main manuscript). In the Visayas, none of the infections were found to be significantly associated with the prevalence of functional illiteracy (Table 6 in main manuscript). In Mindanao, prevalence of *T. trichiura* monoinfection and *P. vivax* infection were positively and significantly associated with prevalence of functional illiteracy (Table 6 in main manuscript). We also found that prevalence of *T. trichiura* moderate/high infection intensity class and the prevalence of *P. vivax* infection were positively and significantly associated with functional illiteracy (Table 7 in main manuscript).

References

1. StataCorp. Stata Statistical Software: Release 13 [Document on the Internet]. College Station: StataCorp LP; 2013 [cited 2014 September 17]. Available from: https://www.stata.com.
