# Supplementary material for: Functional illiteracy burden in soil-transmitted helminth (STH) endemic regions of the Philippines: An ecological study and geographical prediction for 2017
Source: PLoS Negl Trop Dis. 2019 Jun 21;13(6):e0007494. doi: 10.1371/journal.pntd.0007494 (PMC6588226; doi:10.1371/journal.pntd.0007494)
Supplement: S4 Text — (PDF) [file pntd.0007494.s004.pdf]

S4 Text. Estimation of the Population Attributable Fraction (PAF)

We calculated population attributable fractions (PAF) using standard formula to quantify the contribution of risk factors to functional illiteracy. We reported the PAF only for those predictor variables that were found positively and significantly associated with the prevalence of functional illiteracy in our multinomial logistic regression models. For instance, we estimated the PAF of functional illiteracy explained by *P. vivax* infection, and *T. trichiura* monoinfection, separately from Model 5. we used the following standard formula:

$$PAF_1 = \frac{P_1(RRR_1 - 1)}{P_1(RRR_1 - 1) + 1}$$

where P_1 is the mean prevalence of one parasite in the 10 – 19 age group, and RRR_1 is the prevalence-specific ratios of relative risks (RRR).
