# Supplementary material for: Functional illiteracy burden in soil-transmitted helminth (STH) endemic regions of the Philippines: An ecological study and geographical prediction for 2017
Source: PLoS Negl Trop Dis. 2019 Jun 21;13(6):e0007494. doi: 10.1371/journal.pntd.0007494 (PMC6588226; doi:10.1371/journal.pntd.0007494)
Supplement: S5 Text — (PDF) [file pntd.0007494.s005.pdf]

S5 Text. Model specification

For all models, the prior for the intercept (α), and the effect size of covariates beta *(β)* (normal distribution prior) had mean zero and precision 0.01. The geostatistical random effects were assumed to follow a normal distribution, with a mean of zero and a variance of one/tau, where the precision tau was given a gamma prior distribution with shape and scale parameters = 0.001, 0.001. The parameter Phi(Φ) refers to the rate of decay of spatial autocorrelation per unit distance, and indicates the size of clusters. The prior distribution of Φ was uniform with upper and lower bounds set at 0.1 and 100 [1]. The radius of a cluster measured in decimal degrees corresponds to $3/\Phi$. One decimal degree is equivalent to approximately 111 km at the equator (the radii of cluster = $3/\Phi \times 111$km - the further away from the equator the multiplier increases). Bayesian model outputs for parameters of interest and for predictions at unsampled locations are probability distributions, termed posterior distributions, which represent the probability of a variable of interest taking each of a range of plausible values [2]. The posterior distributions can be summarized by statistics such as the posterior mean and 95% Bayesian credible interval (BCI). For model coefficients, a variable was considered as influencing the outcome if it excluded zero. We use marginal prediction using *spatial.unipred* command, which implements independent simulations that do not consider neighbouring values. For each model, a burn-in of 35,000 Markov Chain Monte Carlo iterations was used followed by 5,000 iterations during which values for the intercept and coefficients were stored for parameter estimation and generation of predictive maps. Diagnostic tests for convergence of the stored variables were assessed using visualization of history and density plots of the series of posterior values.

References

1. Soares Magalhães RJ, Biritwum NK, Gyapong JO, Brooker S, Zhang Y, Blair L, et al. Mapping helminth co-infection and co-intensity: geostatistical prediction in ghana. PLoS Negl Trop Dis. 2011;5(6):e1200.
2. Soares Magalhães RJ, Clements AC, Patil AP, Gething PW, Brooker S. The applications of model-based geostatistics in helminth epidemiology and control. Adv Parasitol. 2011;74:267-96.
