# Supplementary material for: Functional illiteracy burden in soil-transmitted helminth (STH) endemic regions of the Philippines: An ecological study and geographical prediction for 2017
Source: PLoS Negl Trop Dis. 2019 Jun 21;13(6):e0007494. doi: 10.1371/journal.pntd.0007494 (PMC6588226; doi:10.1371/journal.pntd.0007494)
Supplement: S6 Text — (PDF) [file pntd.0007494.s006.pdf]

S6 Text. Prediction maps and standard deviation maps

Predicted mean and standard deviation from the prediction models were extracted and mapped using inverse distance weighing (IDW) interpolation tools to generate our prediction map at the nodes of a 0.005 × 0.005 decimal degree grid (approximately $500 m^2$) in ArcGIS. Predicted prevalence of functional illiteracy was categorised into 11 categories: <1%, 1 – $1.\overline{9}$%, 2 – $2.\overline{9}$%, 3 – $3.\overline{9}$%, 4 – $4.\overline{9}$%, 5 – $5.\overline{9}$%, 6 – $6.\overline{9}$%, 7 – $7.\overline{9}$%, 8 – $8.\overline{9}$%, 9 – $9.\overline{9}$%, and ≥10%. Similarly, prediction uncertainty was defined by the standard deviation (SD) and was categorised into five categories: very low uncertainty (SD <0.010), low uncertainty (SD 0.010 – $0.02\overline{9}$), low to moderate uncertainty (SD 0.030 – $0.04\overline{9}$), moderate uncertainty (SD 0.050 – $0.0\overline{9}$), and high uncertainty (SD ≥0.100).
