# Supplementary material for: Functional illiteracy burden in soil-transmitted helminth (STH) endemic regions of the Philippines: An ecological study and geographical prediction for 2017
Source: PLoS Negl Trop Dis. 2019 Jun 21;13(6):e0007494. doi: 10.1371/journal.pntd.0007494 (PMC6588226; doi:10.1371/journal.pntd.0007494)
Supplement: S7 Text — (PDF) [file pntd.0007494.s007.pdf]

S7 Text. Estimation of the number of school-aged children in each functional literacy class in the Philippines in 2017

To estimate the number of children with different levels of functional literacy across the Philippines in 2017, we multiplied each of the predictive raster maps of functional literacy indicators by a raster map of the estimated total number of school-aged children (people per square kilometre) in 2017. To derive the 2017 population raster map for the population aged 10 – 19 years, we multiplied the 2015 population raster map from the AsiaPop project by the reported UNDP annual population growth rate for 2015 to 2020, obtained from the World Population Prospects 2015 Revision Population Database [1-4]. To generate a raster map of the estimated total number of school-aged children in 2017 in the Philippines, the 2017 population raster map was multiplied by the proportion of the Filipino population aged 10 – 19 years to derive a map of the number of school-aged children per square kilometre, and then summed by region. All estimates were conducted in the ArcGIS Map algebra raster calculator [5].

References

1. Gaughan AE, Stevens FR, Linard C, Jia P, Tatem AJ. High resolution population distribution maps for Southeast Asia in 2010 and 2015. PLoS One. 2013;8(2):e55882.
2. Stevens FR, Gaughan AE, Linard C, Tatem AJ. Disaggregating census data for population mapping using random forests with remotely-sensed and ancillary data. PLoS One. 2015;10(2):e0107042.
3. Population Division of the Department of Economic, Social Affairs of the United Nations Secretariat. World Population Prospects 2015 Revision Population Database [Document on the Internet]. New York: United Nations; 2015 [cited 2015 November 26]. Available from: http://esa.un.org/unpd/wpp/unpp/panel_population.htm.
4. Centre for International Earth Science Information Network, Centro Internacional de Agricultura Tropical. Gridded Population of the World (GPW), Version 3 [Document on the Internet]. Palisades: NASA Socioeconomic Data and Applications Center; 2005 [cited 2014 October 1]. Available from: http://sedac.ciesin.columbia.edu/data/set/gpw-v3-population-density.
5. Environmental Systems Research Institute. ArcGIS 10.4 for Desktop [Document on the Internet]. Redlands, CA: Environmental Systems Research Institute; 2015 [cited 2016 January 13]. Available from: http://www.esri.com/.
