# Supplementary figures and images for: Functional illiteracy burden in soil-transmitted helminth (STH) endemic regions of the Philippines: An ecological study and geographical prediction for 2017
Source: PLoS Negl Trop Dis. 2019 Jun 21;13(6):e0007494. doi: 10.1371/journal.pntd.0007494 (PMC6588226; doi:10.1371/journal.pntd.0007494)

**Legend**

- 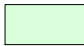 Luzon
- 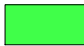 The Visayas
- 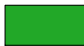 Mindanao
- 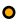 Locations of Survey

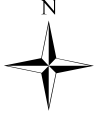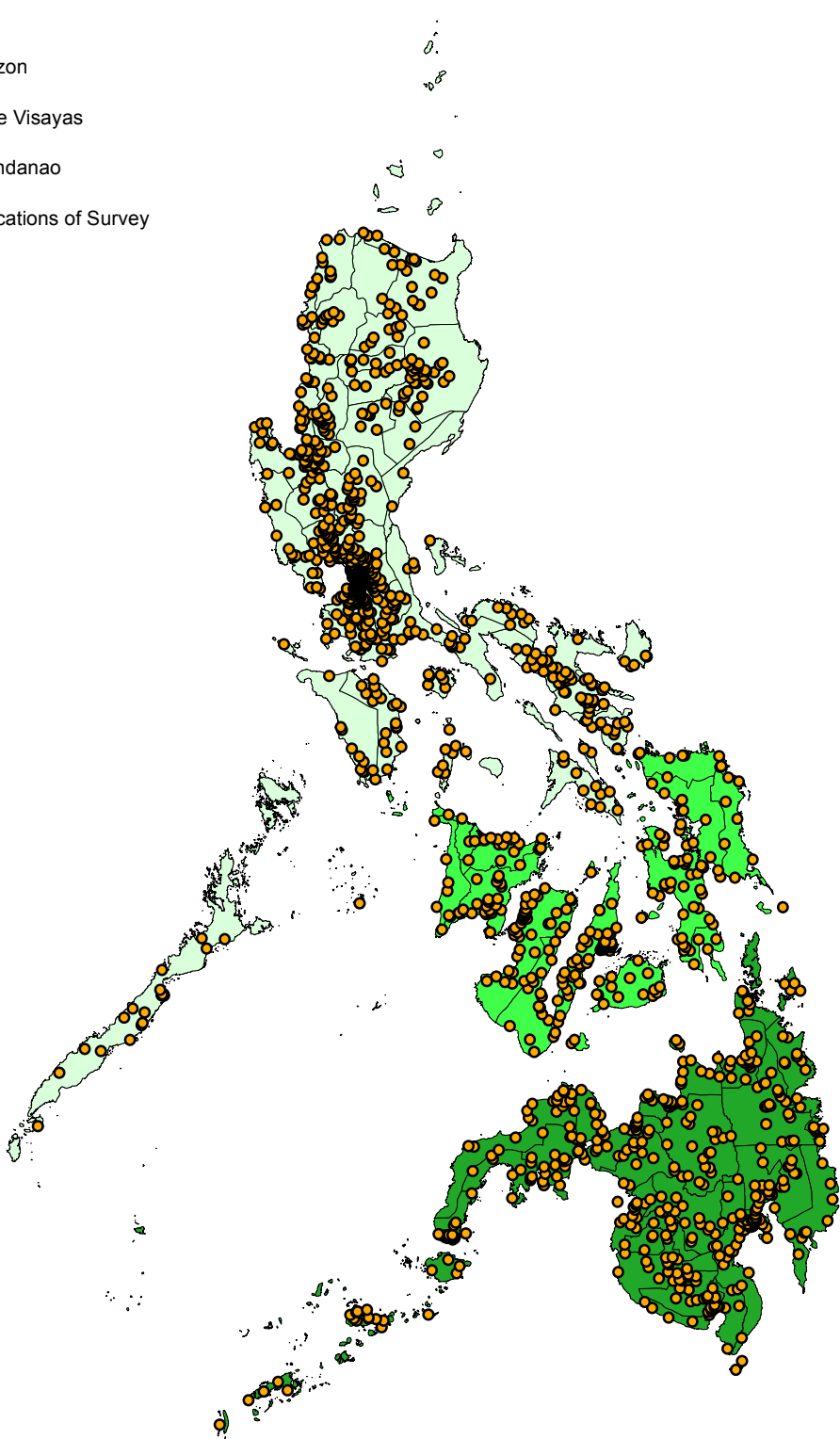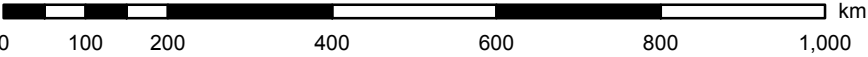

Supplement: S1 Fig — Note: Figure produced by authors of this paper and previously published in Int J Environ Res Public Health [19] and reused under CC BY license. (PDF) [file pntd.0007494.s008.pdf]

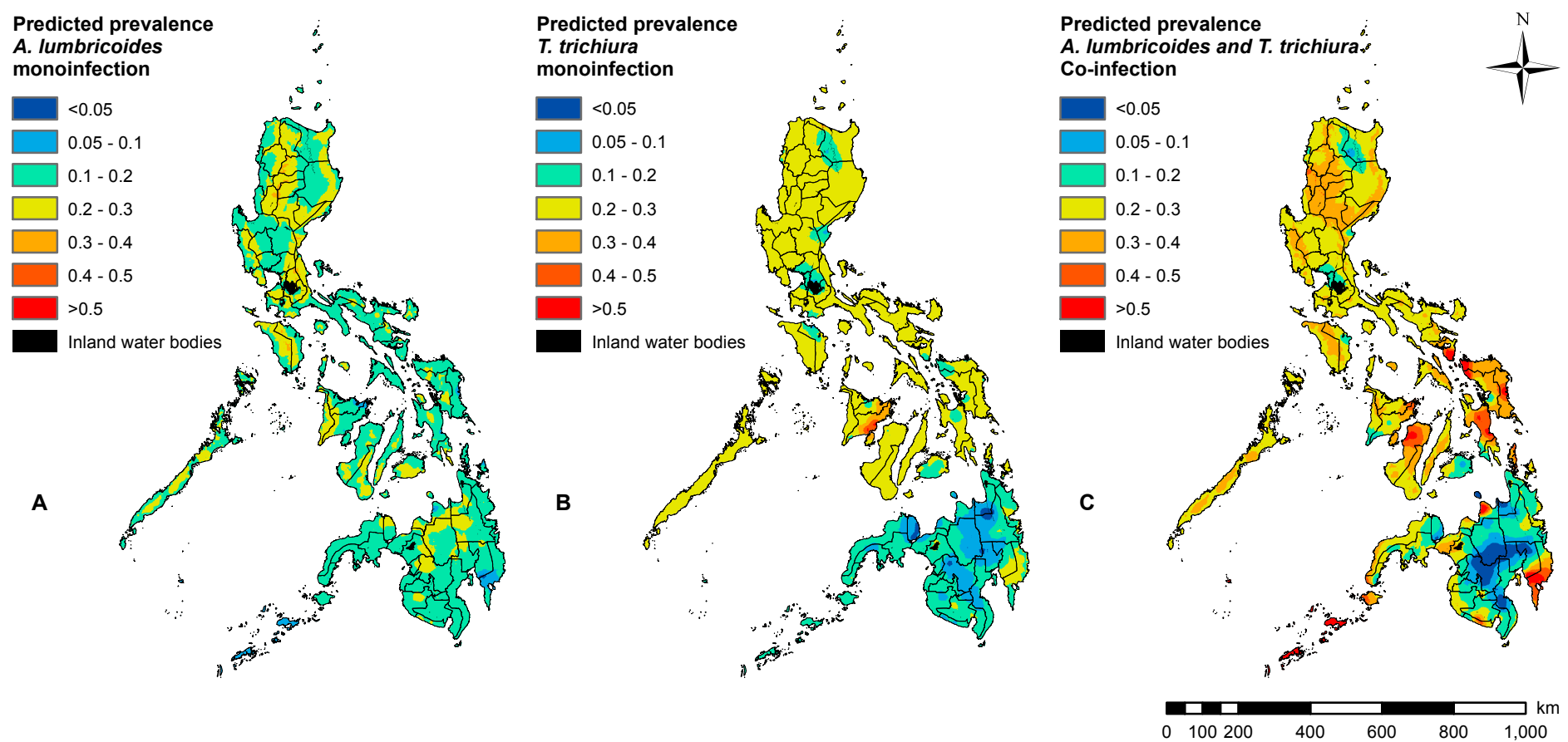

Supplement: S4 Fig — (A) A. lumbricoides monoinfection. (B) T. trichiura monoinfection. (C) A. lumbricoides and T. trichiura co-infection. Note: Figure produced by authors of this paper and previously published in Parasit and Vectors [21] and reused under CC BY license. (PDF) [file pntd.0007494.s011.pdf]

| Region      | Observed                                                                           | Model 1                                                                            | Model 2                                                                             | Model 3                                                                              | Model 4                                                                              | Model 5                                                                              |
|-------------|------------------------------------------------------------------------------------|------------------------------------------------------------------------------------|-------------------------------------------------------------------------------------|--------------------------------------------------------------------------------------|--------------------------------------------------------------------------------------|--------------------------------------------------------------------------------------|
| Luzon       | 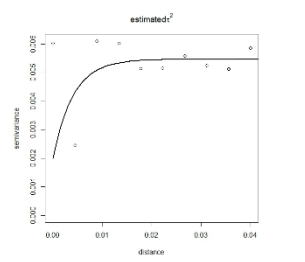  | 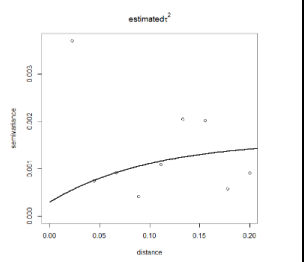  | 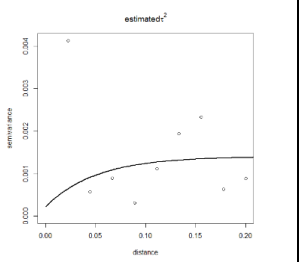  | 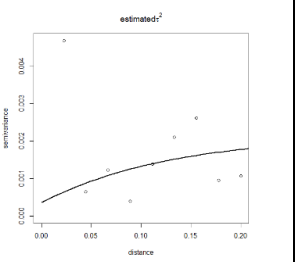  | 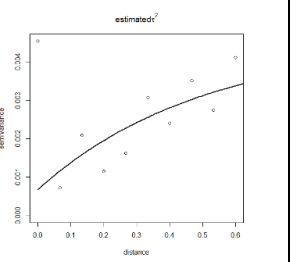  | 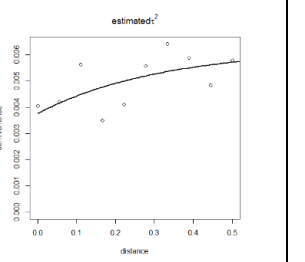  |
| The Visayas | 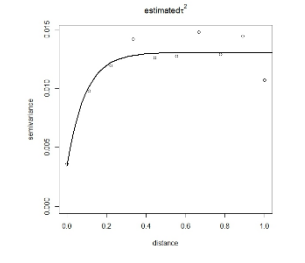  | N/A                                                                                | 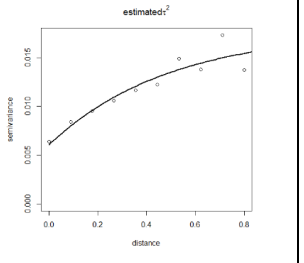  | 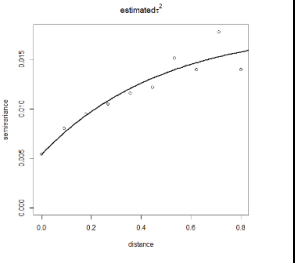  | 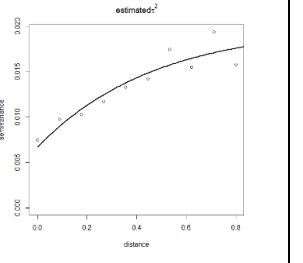  | 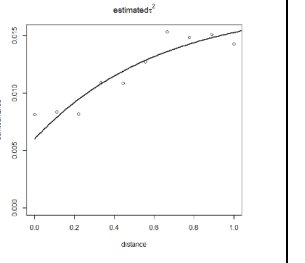  |
| Mindanao    | 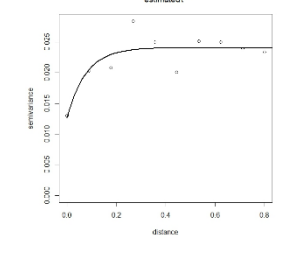 | 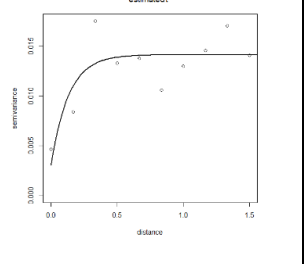 | 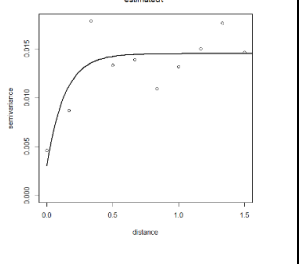 | 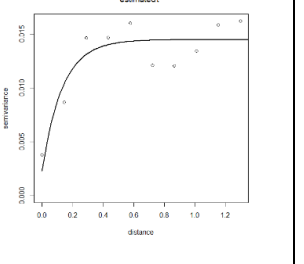 | 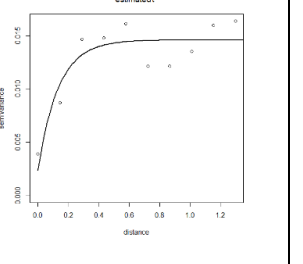 | 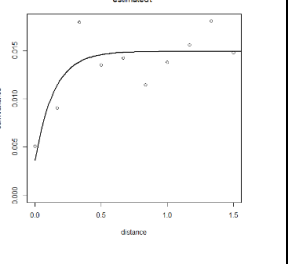 |

Supplement: S6 Fig — Note: Semivariograms of prevalence of observed functional illiteracy indicators and residuals for the final multinomial models (residual semivariograms) at each region to examine the presence of spatial autocorrelation (in decimal degrees). Above semivariograms indicated residual spatial dependency in the prevalence of moderate and low functional literacy indicators in Luzon. In the Visayas, after adjusting for the covariates, residual spatial dependency of functional illiteracy was no longer evident in the semivariograms. In Mindanao, our results indicated residual spatial dependency in the prevalence of moderate functional literacy and functional illiteracy. (PDF) [file pntd.0007494.s013.pdf]
