# Supplementary material for: Functional illiteracy burden in soil-transmitted helminth (STH) endemic regions of the Philippines: An ecological study and geographical prediction for 2017
Source: PLoS Negl Trop Dis. 2019 Jun 21;13(6):e0007494. doi: 10.1371/journal.pntd.0007494 (PMC6588226; doi:10.1371/journal.pntd.0007494)
Supplement: S2 Fig — (A) Plasmodium falciparum. (B) Plasmodium vivax. Note: PfPR2-10 = P. falciparum parasite rate in the 2 to 10 years; PvPR2-10 = P. vivax parasite rate in the 2 to 10 years; Areas with no colours indicate predominantly P. falciparum and P. vivax free areas [20, 40]. (PDF) [file pntd.0007494.s009.pdf]

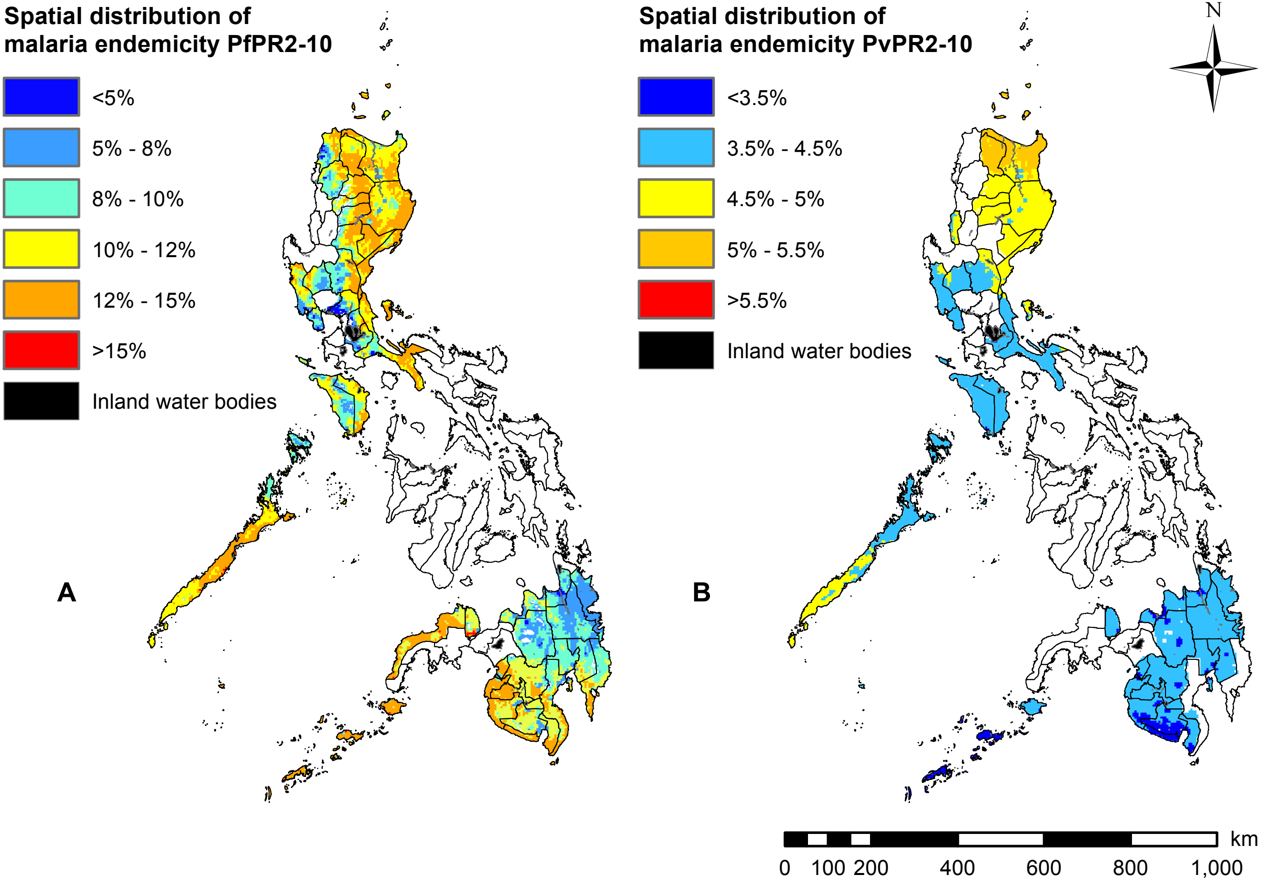
Spatial distribution of malaria endemicity PfPR2-10
<5%
5% - 8%
8% - 10%
10% - 12%
12% - 15%
>15%
Inland water bodies
A
Spatial distribution of malaria endemicity PvPR2-10
<3.5%
3.5% - 4.5%
4.5% - 5%
5% - 5.5%
>5.5%
Inland water bodies
B
N
0 100 200 400 600 800 1,000 km
