# Supplementary material for: Functional illiteracy burden in soil-transmitted helminth (STH) endemic regions of the Philippines: An ecological study and geographical prediction for 2017
Source: PLoS Negl Trop Dis. 2019 Jun 21;13(6):e0007494. doi: 10.1371/journal.pntd.0007494 (PMC6588226; doi:10.1371/journal.pntd.0007494)
Supplement: S3 Fig — (A) A. lumbricoides. (B) T. trichiura. (C) Hookworm. Note: Figure produced by authors of this paper and previously published in PLoS Negl Trop Dis [12] and reused under CC BY license. (PDF) [file pntd.0007494.s010.pdf]

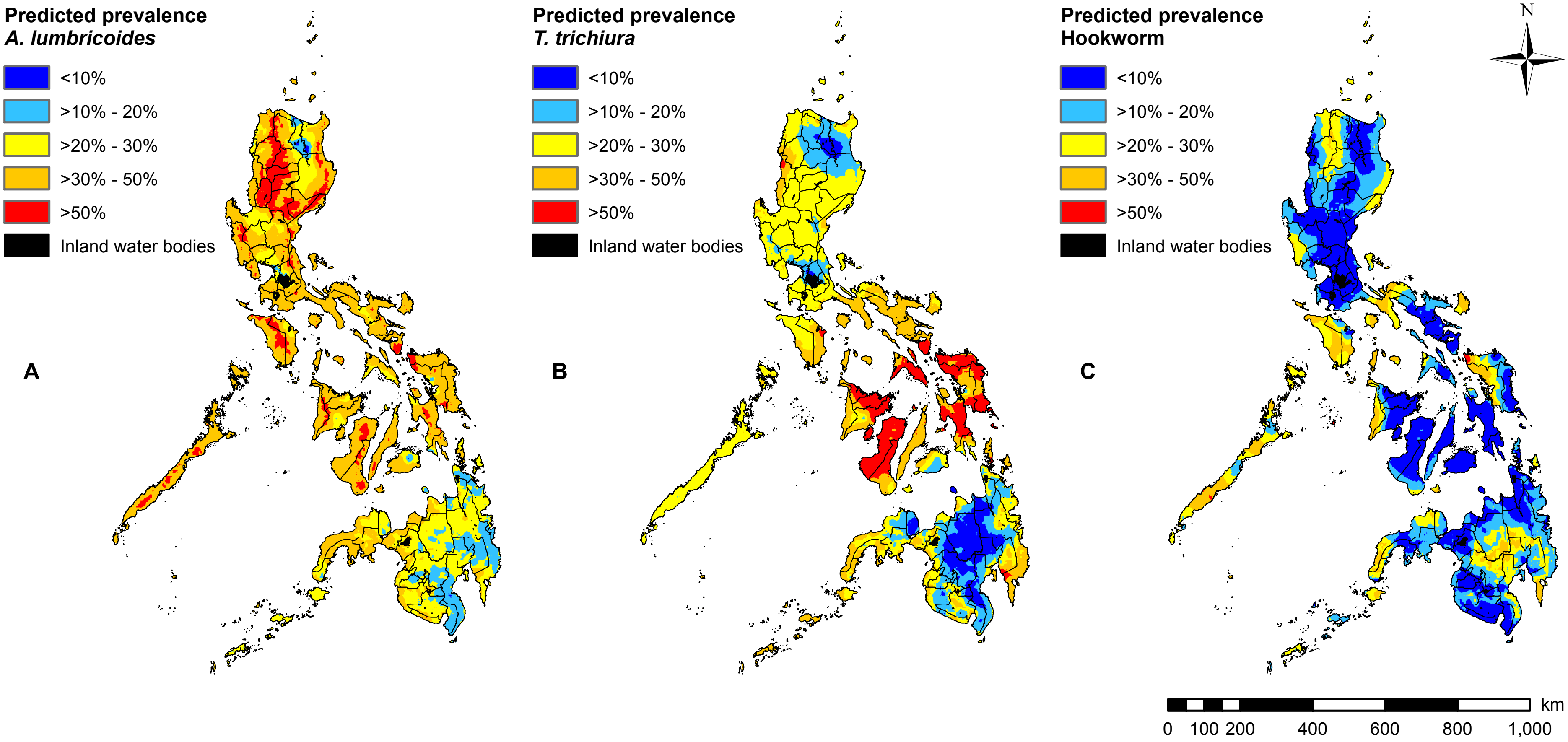

Predicted prevalence
A. lumbricoides
<10%
>10% - 20%
>20% - 30%
>30% - 50%
>50%
Inland water bodies
A
Predicted prevalence
T. trichiura
<10%
>10% - 20%
>20% - 30%
>30% - 50%
>50%
Inland water bodies
B
Predicted prevalence
Hookworm
<10%
>10% - 20%
>20% - 30%
>30% - 50%
>50%
Inland water bodies
C
N
km
0 100 200 400 600 800 1,000
