# Supplementary material for: Functional illiteracy burden in soil-transmitted helminth (STH) endemic regions of the Philippines: An ecological study and geographical prediction for 2017
Source: PLoS Negl Trop Dis. 2019 Jun 21;13(6):e0007494. doi: 10.1371/journal.pntd.0007494 (PMC6588226; doi:10.1371/journal.pntd.0007494)
Supplement: S5 Fig — Note: A higher number of households in Mindanao were accessing unprotected wells (11.7% in Mindanao compared to 5% in Luzon and 7% in the Visayas), and had access to either closed-pit or open-pit latrines compared to the Visayas and Luzon (18% in Mindanao compared to 6.7%, 5.2%, respectively). A higher proportion of households in the Visayas were practising open-defecation compared to Luzon and Mindanao (12.5%, 4.7% and 6.4%, respectively). Figure produced by authors of this paper and previously published in Int J Environ Res Public Health [19] and reused under CC BY license. (PDF) [file pntd.0007494.s012.pdf]

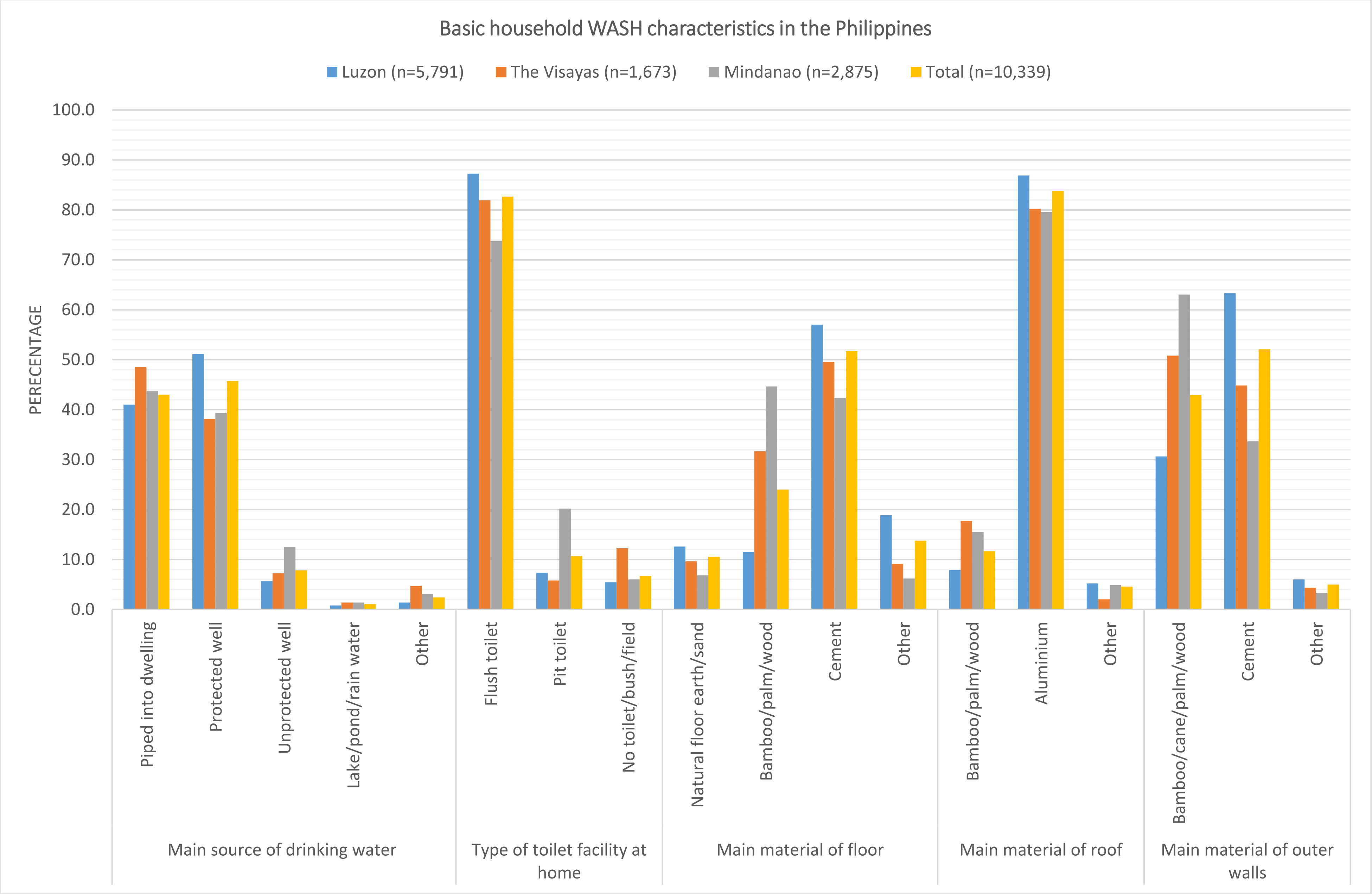

Basic household WASH characteristics in the Philippines
Luzon (n=5,791)
The Visayas (n=1,673)
Mindanao (n=2,875)
Total (n=10,339)
PERECENTAGE
100.0
90.0
80.0
70.0
60.0
50.0
40.0
30.0
20.0
10.0
0.0
Piped into dwelling
Protected well
Unprotected well
Lake/pond/rain water
Other
Flush toilet
Pit toilet
No toilet/bush/field
Natural floor earth/sand
Bamboo/palm/wood
Cement
Other
Bamboo/palm/wood
Aluminium
Other
Bamboo/cane/palm/wood
Cement
Other
Main source of drinking water
Type of toilet facility at home
Main material of floor
Main material of roof
Main material of outer walls
