# Supplementary material for: Functional illiteracy burden in soil-transmitted helminth (STH) endemic regions of the Philippines: An ecological study and geographical prediction for 2017
Source: PLoS Negl Trop Dis. 2019 Jun 21;13(6):e0007494. doi: 10.1371/journal.pntd.0007494 (PMC6588226; doi:10.1371/journal.pntd.0007494)
Supplement: S7 Fig — (A) Moderate functional literacy. (B) Low functional literacy. Note: Results based on Model 3 for Luzon and the Visayas; based on Model 5 for Mindanao. (PDF) [file pntd.0007494.s014.pdf]

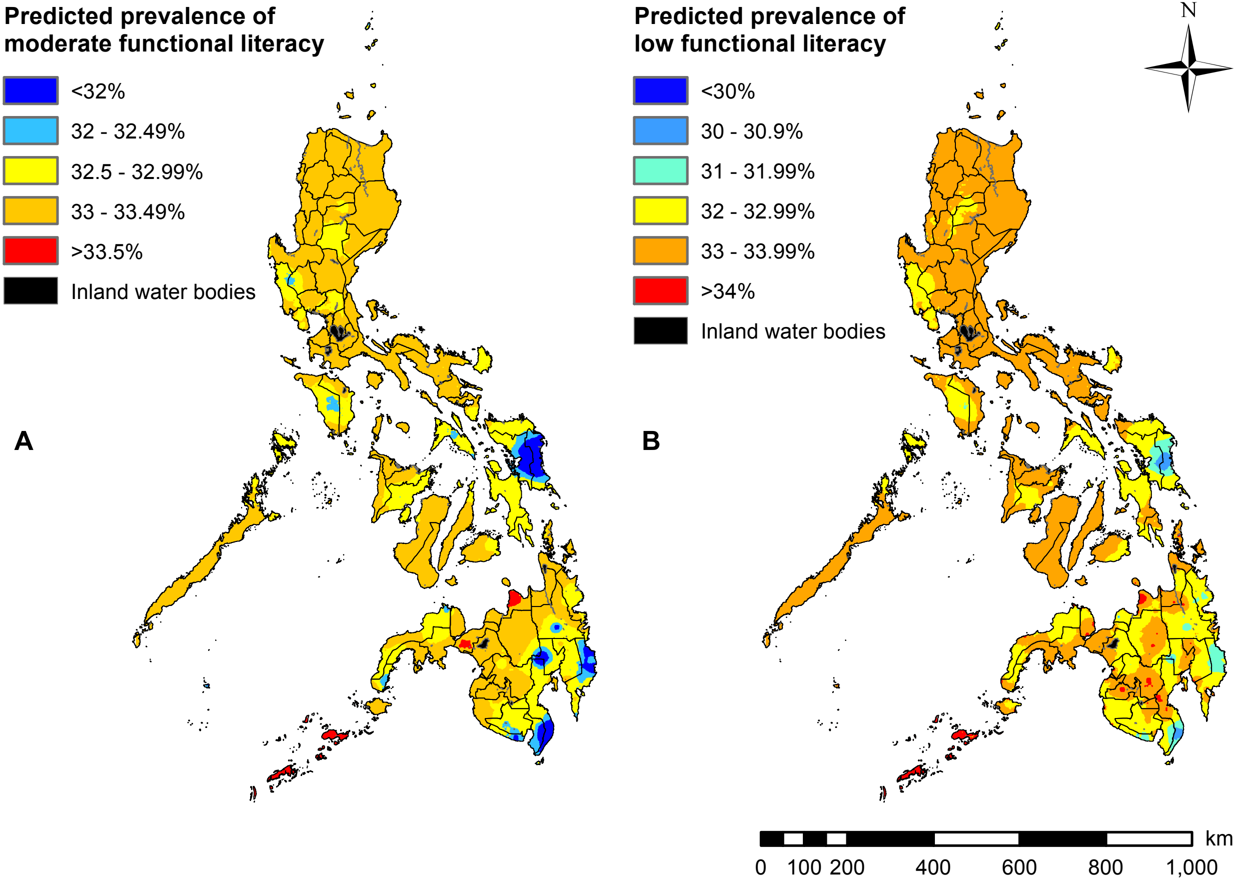

Predicted prevalence of moderate functional literacy
<32%
32 - 32.49%
32.5 - 32.99%
33 - 33.49%
>33.5%
Inland water bodies
A
Predicted prevalence of low functional literacy
<30%
30 - 30.9%
31 - 31.99%
32 - 32.99%
33 - 33.99%
>34%
Inland water bodies
B
N
km
0 100 200 400 600 800 1,000
