# Supplementary material for: Functional illiteracy burden in soil-transmitted helminth (STH) endemic regions of the Philippines: An ecological study and geographical prediction for 2017
Source: PLoS Negl Trop Dis. 2019 Jun 21;13(6):e0007494. doi: 10.1371/journal.pntd.0007494 (PMC6588226; doi:10.1371/journal.pntd.0007494)
Supplement: S8 Fig — (A) Luzon. (B) The Visayas. (C) Mindanao. Note: Results based on Model 3 for Luzon and the Visayas; based on Model 5 for Mindanao. (PDF) [file pntd.0007494.s015.pdf]

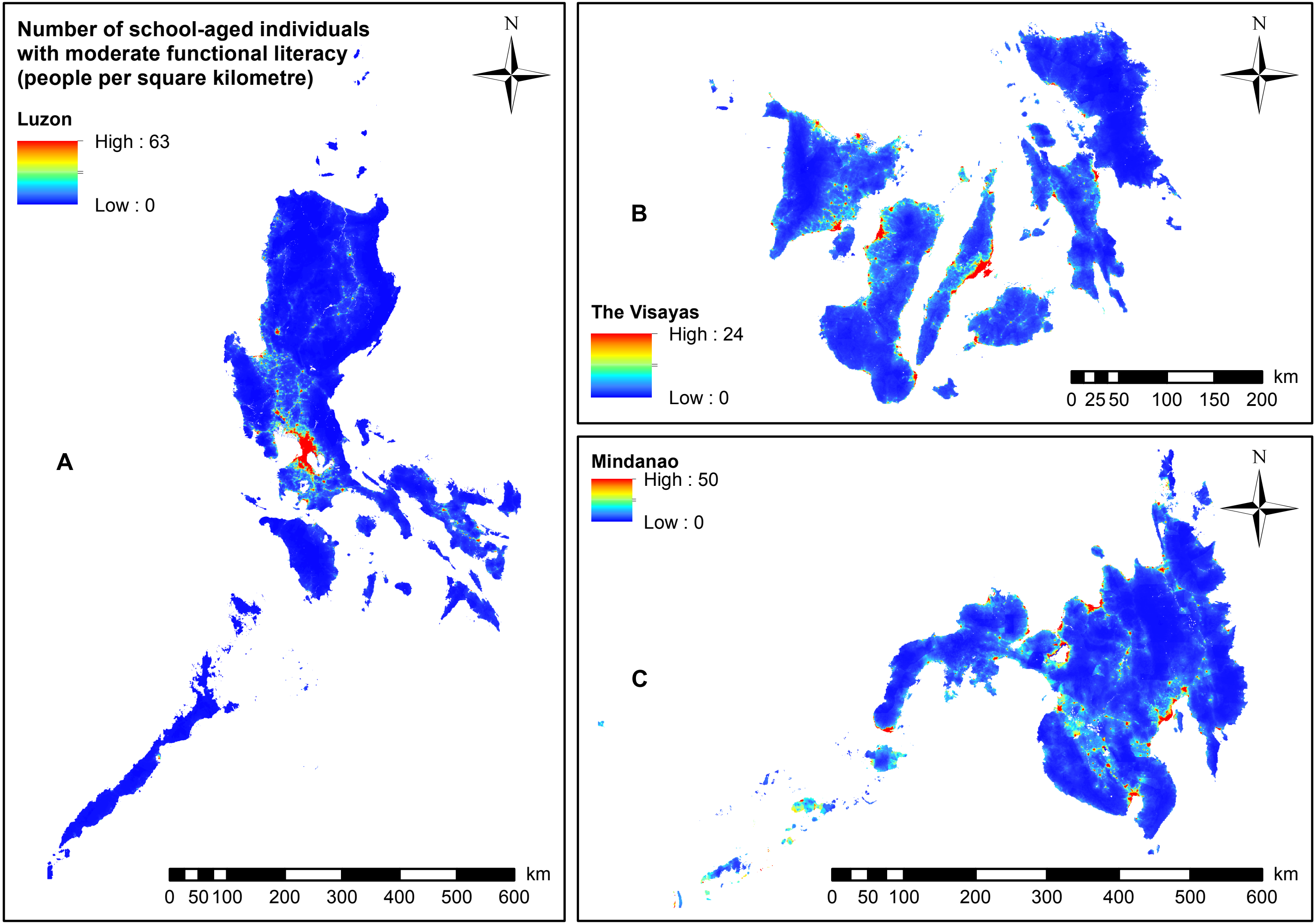
Number of school-aged individuals
with moderate functional literacy
(people per square kilometre)
N
Luzon
High : 63
Low : 0
A
km
0 50 100 200 300 400 500 600
N
B
The Visayas
High : 24
Low : 0
km
0 25 50 100 150 200
Mindanao
High : 50
Low : 0
N
C
km
0 50 100 200 300 400 500 600
