# Supplementary material for: Functional illiteracy burden in soil-transmitted helminth (STH) endemic regions of the Philippines: An ecological study and geographical prediction for 2017
Source: PLoS Negl Trop Dis. 2019 Jun 21;13(6):e0007494. doi: 10.1371/journal.pntd.0007494 (PMC6588226; doi:10.1371/journal.pntd.0007494)
Supplement: S9 Fig — (PDF) [file pntd.0007494.s016.pdf]

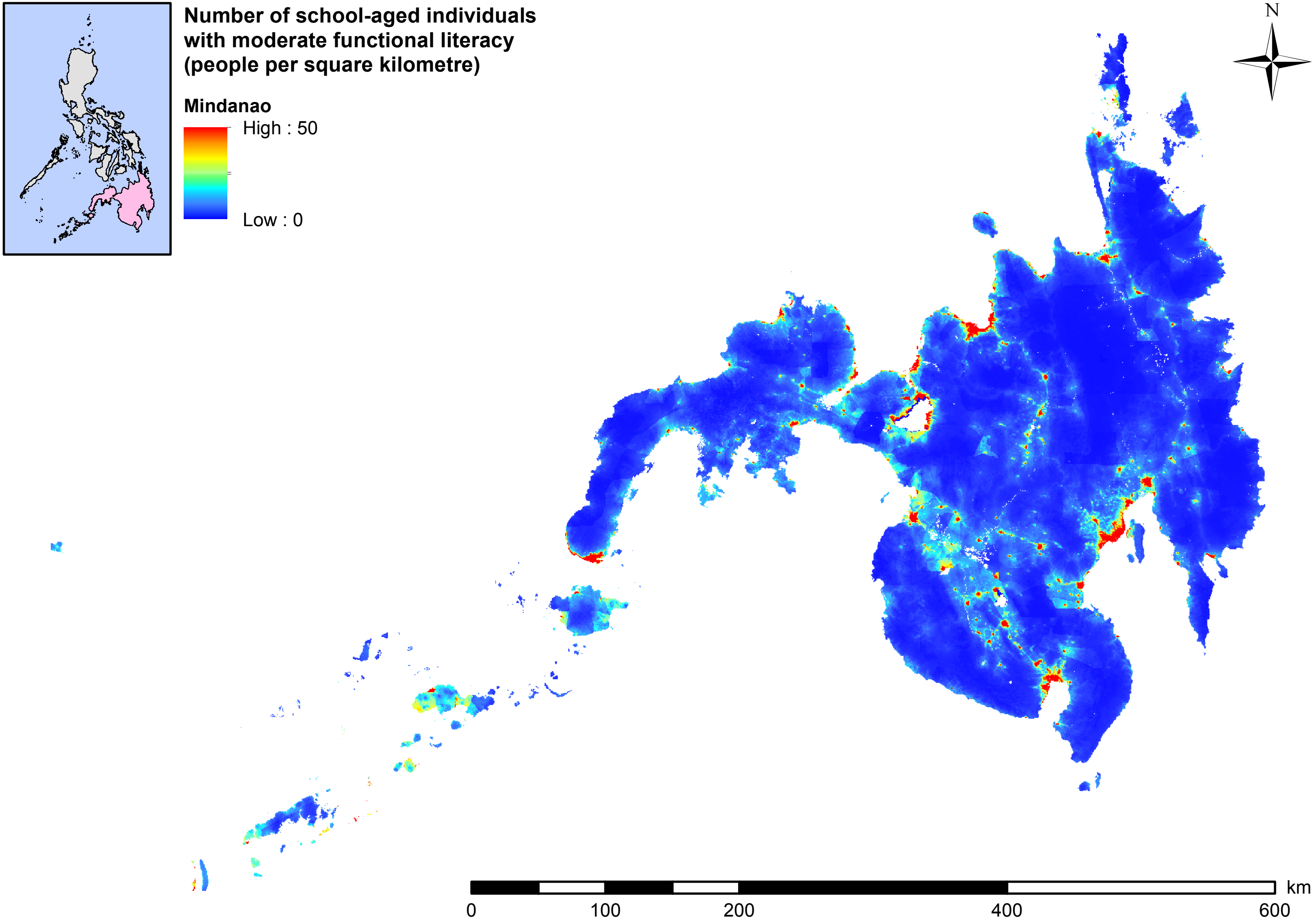
Number of school-aged individuals
with moderate functional literacy
(people per square kilometre)
Mindanao
High : 50
Low : 0
N
km
0
100
200
400
600
