# Supplementary material for: Functional illiteracy burden in soil-transmitted helminth (STH) endemic regions of the Philippines: An ecological study and geographical prediction for 2017
Source: PLoS Negl Trop Dis. 2019 Jun 21;13(6):e0007494. doi: 10.1371/journal.pntd.0007494 (PMC6588226; doi:10.1371/journal.pntd.0007494)
Supplement: S1 Table — (PDF) [file pntd.0007494.s017.pdf]

**Cognitive stimulation sub-index (Based on FLEMMS item number)**	**Yes (1) / No (0)**
Is there a TV at home?	
Is there a phone at home?	
Is there a radio at home?	
Is there a CD at home?	
Is there a karaoke machine at home?	
Is there a personal computer at home?	
Does your family read newspapers?	
Does your family read the magazines?	
Does your family read the posters?	
Does your family watch TV?	
Does your family listen to radio?	
Does your family watch movies?	
Does your family use internet?	
Does your family attend meetings of organisations?	
Does your family own a boat?	
Does your family own a tractor?	
Does your family own a car?	
Does your family own a tricycle?	
Does your family own a bicycle?	
Total score (/19)	
