# Supplementary material for: Functional illiteracy burden in soil-transmitted helminth (STH) endemic regions of the Philippines: An ecological study and geographical prediction for 2017
Source: PLoS Negl Trop Dis. 2019 Jun 21;13(6):e0007494. doi: 10.1371/journal.pntd.0007494 (PMC6588226; doi:10.1371/journal.pntd.0007494)
Supplement: S2 Table — (PDF) [file pntd.0007494.s018.pdf]

Variables		Regions		
		**Luzon (n=5,791)**	**The Visayas (n=1,673)**	**Mindanao (n=2,875)**
Age	Average age (Standard deviation)	42.1 (12.2)	42.2 (12.2)	39.9 (12.2)
Sex	Male	2,900 (50.1)	910 (54.4)	1,499 (52.1)
	Female	2,891 (49.9)	763 (45.6)	1,376 (47.9)
Marital status	Single	842 (14.5)	286 (17.1)	487 (16.9)
	Married	4,454 (76.9)	1,286 (75.8)	2,210 (76.9)
	Widow	349 (6.0)	95 (5.7)	130 (4.5)
	Divorced	146 (2.5)	24 (1.4)	48 (1.7)
Functional literacy level	Functional literate	2,979 (51.4)	582 (35)	1,075 (37.4)
	Moderate functional literate	1,940 (33.5)	664 (40.0)	1,138 (39.6)
	Low functional literate	520 (9.0)	180 (10.0)	232 (8.0)
	Functional illiterate	352 (6.1)	247 (15.0)	430 (15.0)
Highest education attainment	No grade completed	82 (1.4)	56 (3.4)	197 (6.9)
	Elementary school	3,755 (64.8)	1,160 (69.3)	1,702 (59.2)
	High school level or higher	1,954 (33.7)	457 (27.3)	976 (33.9)
Occupation	Worked for private household	1787 (30.8)	485 (29)	707 (24.6)
	Worked for government	94 (1.6)	33 (2.0)	72 (2.5)
	Worked with pay on own family-operated farm or business	1,890 (32.6)	647 (38.7)	1,037 (36.1)
	Worked without pay on own family-operated farm or business	544 (9.4)	189 (11.3)	395 (13.7)
	Unemployed	1,476 (25.6)	319 (19.0)	664 (23.1)

Note: Unless otherwise indicated, values represent the absolute number followed by the percentage within parentheses.
