# Supplementary material for: Functional illiteracy burden in soil-transmitted helminth (STH) endemic regions of the Philippines: An ecological study and geographical prediction for 2017
Source: PLoS Negl Trop Dis. 2019 Jun 21;13(6):e0007494. doi: 10.1371/journal.pntd.0007494 (PMC6588226; doi:10.1371/journal.pntd.0007494)
Supplement: S3 Table — (PDF) [file pntd.0007494.s019.pdf]

Socioeconomic status (SES)	Region			Total
	Luzon	The Visayas	Mindanao	
Poor<br>(Low SES)	1,925<br>(33.2)	713<br>(42.6)	1,434<br>(49.9)	4,072<br>(39.4)
Non-poor<br>(High SES)	3,866<br>(66.8)	960<br>(57.4)	1,441<br>(50.1)	6,267<br>(60.6)
Total	5,791<br>(100)	1,673<br>(100)	2,875<br>(100)	10,339<br>(100)

Note: Unless otherwise indicated, values represent the absolute number followed by the percentage within parentheses.
