# Supplementary material for: Functional illiteracy burden in soil-transmitted helminth (STH) endemic regions of the Philippines: An ecological study and geographical prediction for 2017
Source: PLoS Negl Trop Dis. 2019 Jun 21;13(6):e0007494. doi: 10.1371/journal.pntd.0007494 (PMC6588226; doi:10.1371/journal.pntd.0007494)
Supplement: S4 Table — (PDF) [file pntd.0007494.s020.pdf]

Household education stimuli score	Region		
	Luzon (n=5,791)	The Visayas (n=1,673)	Mindanao (n=2,875)
Average score	8.12	7.56	7.03
Standard Deviation (SD)	3.28	3.14	3.33
95% Confidence Interval	8.04, 8.20	7.01, 7.71	6.91, 7.15
